# The cost-effectiveness of risk-stratified breast cancer screening in the UK

**DOI:** 10.1038/s41416-023-02461-1

**Published:** 2023-10-17

**Authors:** Harry Hill, Ben Kearns, Nora Pashayan, Cristina Roadevin, Peter Sasieni, Judith Offman, Stephen Duffy

**Affiliations:** 1 School of Medicine and Population Health, University of Sheffield, Sheffield, England; 2 Lumanity Inc, Sheffield, England; 3 Department of Applied Health Research, University College London, London, England; 4 Nottingham Clinical Trials Unit, University of Nottingham, Nottingham, England; 5 Life Sciences & Medicine, King’s College London, London, England; 6 Wolfson Institute of Population Health, Queen Mary University of London, London, England

**Keywords:** Health care economics, Health policy, Health services, Medical imaging

## Abstract

**Background:**

There has been growing interest in the UK and internationally of risk-stratified breast screening whereby individualised risk assessment may inform screening frequency, starting age, screening instrument used, or even decisions not to screen. This study evaluates the cost-effectiveness of eight proposals for risk-stratified screening regimens compared to both the current UK screening programme and no national screening.

**Methods:**

A person-level microsimulation model was developed to estimate health-related quality of life, cancer survival and NHS costs over the lifetime of the female population eligible for screening in the UK.

**Results:**

Compared with both the current screening programme and no screening, risk-stratified regimens generated additional costs and QALYs, and had a larger net health benefit. The likelihood of the current screening programme being the optimal scenario was less than 1%. No screening amongst the lowest risk group, and triannual, biennial and annual screening amongst the three higher risk groups was the optimal screening strategy from those evaluated.

**Conclusions:**

We found that risk-stratified breast cancer screening has the potential to be beneficial for women at the population level, but the net health benefit will depend on the particular risk-based strategy.

## Background

Breast cancer screening has been implemented in many high-income countries to detect breast cancer at an early stage and thereby decrease breast cancer mortality. The benefit of breast cancer screening is that cancers are detected at an earlier stage. Early detection of cancers is an important policy priority for the UK NHS, which has set a target that, by 2028, the proportion of cancers diagnosed at Stages 1 and 2 will rise from around half (in 2019) to three-quarters of cancer patients [1]. To meet this objective the NHS plans to increase uptake to the breast screening programme, and modernise and expand diagnostic capacity.

Women may be harmed by breast screening from the pain incurred during mammogram screening, the hazard of ionising radiation from mammography, the distress caused by false positive (FP) results and overdiagnosis [2]. Overdiagnosis is defined as the diagnosis of cancer as a result of screening which would not have been diagnosed in the person’s lifetime if screening had not taken place. Risk-stratified breast cancer screening (RSBCS) has the potential to improve the benefit-harm ratio because screening programmes offered to women are tailored to a woman’s assessed risk.

In the United Kingdom (UK) the National Health Service Breast Screening Programme (NHSBSP) is a national population-based organised screening programme that invites all woman from the age of 50 to the age of 70 to breast cancer screening by mammogram once every 3 years [2]. This is an age-based screening approach but UK NHSBSP also offers magnetic resonance imaging (MRI) screening or annual mammogram to women at high-risk of developing breast cancer, women who carry risk-related genes such as *BRCA1* and *BRCA2* [3]. There are other accurate approaches to estimating a woman’s individual risk that cost less than genetic testing to administer, including using breast density derived from mammogram and self-report questions assessing personal and family history. Assessing the adult female population with these alternative methods, and increasing the use of genetic testing, could be used to identify new cases of women who are at high-risk, and consequently improve the NHSBSP’s detection rate of cancers. A consistently accurate model for predicting 10-year breast cancer incidence is the Tyrer–Cuzick (TC) model [4, 5]. This is a self-report questionnaire, which was updated in 2019 to include mammographic density and polygenic risk factors [6].

The aim of this research is to use an economic model to assess if introducing new RSBCS programmes into the UK NHSBSP would improve the health of the women’s population while also being a justifiable use of the NHS budget. The term “model” has different meanings in different settings but typically models are used to provide policymakers with a structured way to make decisions based on quantitative estimates [7]. This is the case in the study because an economic model is used to synthesise evidence from multiple sources and establish policy-relevant outcomes in a scenario where no direct evidence exists, based on clearly stated and justified assumptions and choices of evidence.

## Methods

Eight RSBCS regimens developed by three independent research groups were evaluated: Breast Screening Risk Adaptive Imaging for Density (BRAID) [8], Adapting Breast Cancer Screening Strategy Using Personalised Risk Estimation (ASSURE) [9], and Predicting Risk of Cancer at Screening (PROCAS) [10]. These are described in Table 1. All percentages in Table 1 refer to the 10-year absolute risk of developing cancer. In addition to varying in the frequency of screening by risk group, an important difference between the regimens is the instrument used to assess risk, which is by mammographic breast density alone in BRAID, TC augmented by mammographic breast density in ASSURE and TC augmented by mammographic breast density and genetic profile in PROCAS. In addition, current screening in the NHSBSP and a strategy of no screening are considered.

**Table 1:** Description of RSBCS programmes.

Regimen	Description of risk groupings	Screening programmes offered to woman in the risk groups
BRAID 1	(1) Breast imaging reporting and data system (BI-RADS) classification A&B or (2) BI-RADS classification C&D	(1) Triennial mammogram (MAM), or (2) additional 18-monthly automated ultrasound (AUS)
BRAID 2	(1) BI-RADS A&B or (2) BI-RADS C&D	(1) Triennial MAM, or (2) additional 18-monthly contrast-enhanced spectral MAM (CESM)
BRAID 3	(1) BI-RADS A&B or (2) BI-RADS C&D	(1) Triennial MAM, or (2) additional 18-monthly abbreviated Magnetic Resonance Imaging (AMRI)
ASSURE 1	(1) < 3.5% Tyrer–Cuzick (TC), (2) 3.5% - 8% TC or (3) > 8% TC	(1) Triennial MAM, (2) biennial MAM, or (3) annual MAM
ASSURE 2	(1) Lowest TC risk tertile, (2) middle TC tertile or (3) highest TC risk tertile	(1) Triennial MAM, (2) biennial MAM, or (3) annual MAM
ASSURE 3	(1) Volumetric Breast Density (VBD) 1 & 2, (2) VBD 3 & 4 or (3) VBD 3&4 and >8% TC	(1) Triennial MAM, (2) triennial MAM with handheld ultrasound (HUS), or (3) triennial MAM with MRI
ASSURE 4	(1) < 3.5% TC and VBD 1&2, (2) < 3.5% TC and VBD 3&4, (3) 3.5–8% TC and VBD 1&2, (4) 3.5–8% TC and VBD 3&4, (5) > 8% TC and VBD 1&2 or (6) > 8% TC and VBD 3&4	(1) Triennial MAM, (2) triennial MAM with HUS, (3) biennial MAM, (4) biennial MAM with HUS, (5) annual MAM, or (6) annual MAM with HUS
PROCAS	(1) Age 35–49 y and >2.5% TC, (2) age 45–49 y and >1.5% TC, (3) age 50 and above and < 1.5% TC, (4) age 50 and above and 1.5–2.5% TC, (5) age 50 and above and 2.5–5% TC or (6) age 50 and above and >5% TC	(1) Annual MAM to age 50, (2) annual MAM to age 50, (3) no screening, (4) triennial MAM, (5) biennial MAM, or (6) annual MAM

*BRAID* Breast Screening Risk Adaptive Imaging for Density, *ASSURE* Adapting Breast Cancer Screening Strategy Using Personalised Risk Estimation, *PROCAS* Predicting Risk of Cancer at Screening, *TC* Tyrer–Cuzick, *BI-RADS* breast imaging reporting and data system, *VBD* volumetric breast density, *MAM* mammogram, *AUS* automated ultrasound, *HUS* handheld ultrasound, *CESM* contrast-enhanced spectral mammogram, *AMRI* abbreviated magnetic resonance imaging, *MRI* magnetic resonance imaging.

A health economic analysis plan was developed as part of the project protocol approved by the funder, the Department of Health and Social Care (DHSC), prior to the start of the project. The DHSC had no role in the design, conduct and reporting of the analysis, and the findings of this research may not represent their views. Following the analysis plan, the model type, model structure and parameter information was informed by a review, undertaken for this study, of the national breast cancer screening economic models. This model used is a microsimulation model of clinically important events built in R (version 4.2.2) [11], using the package ‘simmer’. The code of the model is available upon request to the corresponding author. The economic evaluation was undertaken with stakeholder involvement to ensure its relevance. It was designed with the direct assistance of members of the UK National Screening Committee, who advised on the appropriateness of model assumptions, sources used for model parameters and which risk-stratified regimens were policy-relevant and so should be included in the model. The final design of the model was reliant on feedback from patients with breast cancer. In addition, the conduct of the project was overseen by a Steering Committee that included policy stakeholder representatives from the DHSC.

The population examined in the model is 20,000 women who are followed from age of first invitation to a risk assessment to mortality (by cancer or other causes), with invitations to screening appointments (that may or may not be attended) taking place at regular intervals. In brief, the model captures the following pattern of cause and effect from women’s attendance at risk assessments and screening appointments to cancer prognosis. As women become older in the model they are at a higher level of assessed risk, and consequently after a risk assessment they are invited for screening more often (older women are also more likely to attend screening appointments). This shortens the intervals between screening appointments, which leads to a smaller-sized tumour at screening appointments. Smaller tumours are harder to detect at a screening appointment, but if it is detected the tumour is less likely to be at an advanced-stage (since it has had less time to grow between screening appointments). A key reason for using a microsimulation model is that women can have different attendance behaviour, such that not all women will experience the exact pattern of cause and effect just described. For instance, older women are at high-risk and are therefore frequently invited to screening causing tumours to be detected at an earlier stage. However, the model allows some of those older women to not attend screening appointments, and they will have a tumour at a more advanced-stage when it is detected.

**Fig. 1:**
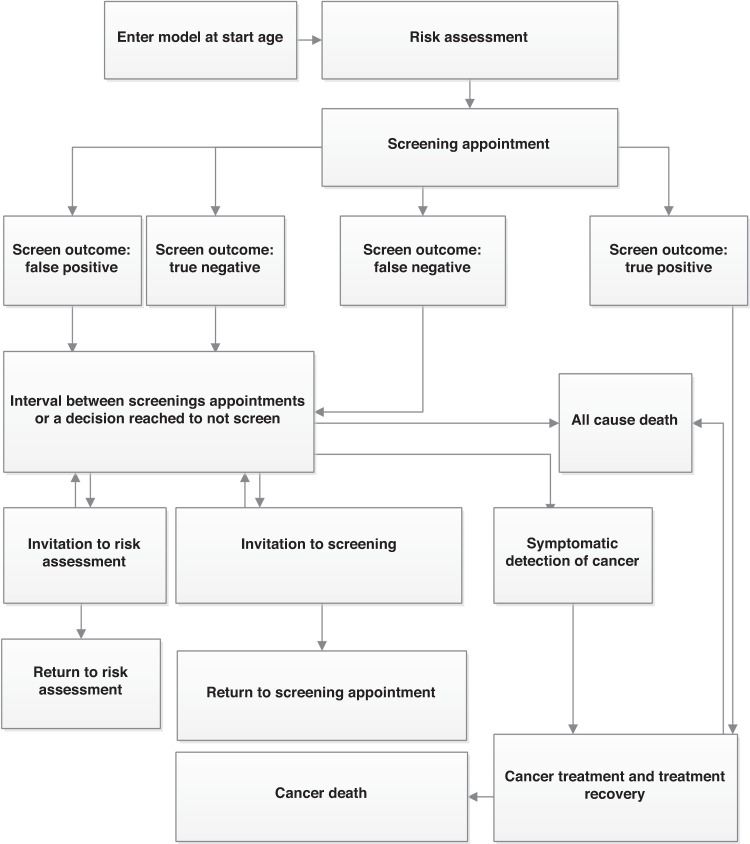
Natural history of cancer. Structure of events in the model.

Figure 1 illustrates the model structure. It shows the ordering of clinical events in the model and hence the possible pathways women can take from risk assessment to screening and from cancer detection to mortality. The arrows in the diagram show the possible clinical events a woman can move to from her current event. Women enter the model in the top left of the figure at an age at which she is first invited to a risk assessment and the next event is when she attends a risk assessment appointment. Women exit the model after reaching the event of death (either by all-cause or cancer mortality). What follows is a detailed description of the information that is being tracked and processed at each event in the model. The events are discussed in the order in which they appear from the top of Fig. 1.

### Model event: model entry

Women enter the model when they are first invited for a risk assessment at age 50 (BRAID, ASSURE) or 35 (PROCAS). Upon entry into the model, every woman is given a predetermined age of death from all causes, and a probability of developing breast cancer during their lifetime, which are both sampled from Office for National Statistics (ONS) UK women life expectancy tables [12] and the breast cancer incidence in England [13]. To simulate the no-screening scenario, observed incidence is reduced by 3.7%, which is the most recent estimate of excess cancer incidence from the NHSBSP [14]. The estimates are age-based and are provided in Appendix Table 1. An age at which the tumour is symptomatically detected in the absence of any screening programme is established from sampling from this age distribution of excess incidence. There is evidence that overdiagnosis is greater with more frequent screening intervals [15]. The overdiagnosis rate for 2-yearly screening compared to no screening is assumed to be 3.85% which is the mean overdiagnosis rate for EU countries with 2-yearly routine screening in Europe, and the same relative increase of incidence is assumed when moving from 2-yearly screening to 1-yearly screening [16]. Details of the methods used to account for overdiagnosis are summarised in the Appendix (Section 2).

### Model event: risk assessment

All women receive at least one mammogram to measure breast density, and the first mammogram takes place upon entry into the model (at an initial risk assessment appointment). A mammogram is used at subsequent NHS risk assessment appointment incurring a mammogram screening cost of £57.69 (further risk assessment and screening costs applied at risk assessment and screening appointments are detailed in the Online Appendix Section 7). Cumulus breast density values are assigned to woman at first screening based on the breast density distribution of UK women [17] for ages 49–54 and women in the People’s Republic of China for ages below 49 years [18] (based on the best available evidence, as summarised in Appendix Table 3). Under BRAID, screening programmes are assigned based on Volpara density values, which are established by transforming Cumulus breast density values based on a published conversion rate [19]. Under PROCAS and ASSURE women have risk measured by an augmented TC risk score sampled from a distribution of scores from women in England that depends on whether the woman develops cancer in the next 10 years [6, 20] (Appendix Table 4). This ensures that the risk scores predict 10-year cancer incidence. To ascertain risk at subsequent risk assessments, Cumulus breast density declines annually based on age and current Cumulus breast density (Appendix Table 5) based on Australian findings [19] and TC score increases annually based on age, estimated from the TC model in UK women [21]. Other risk factors are assumed to not change as women age. Further details on the method of assigning establishing TC change as women age in the model is presented in the Appendix (Section 3).

### Model event: screening appointment

**Fig. 2:**
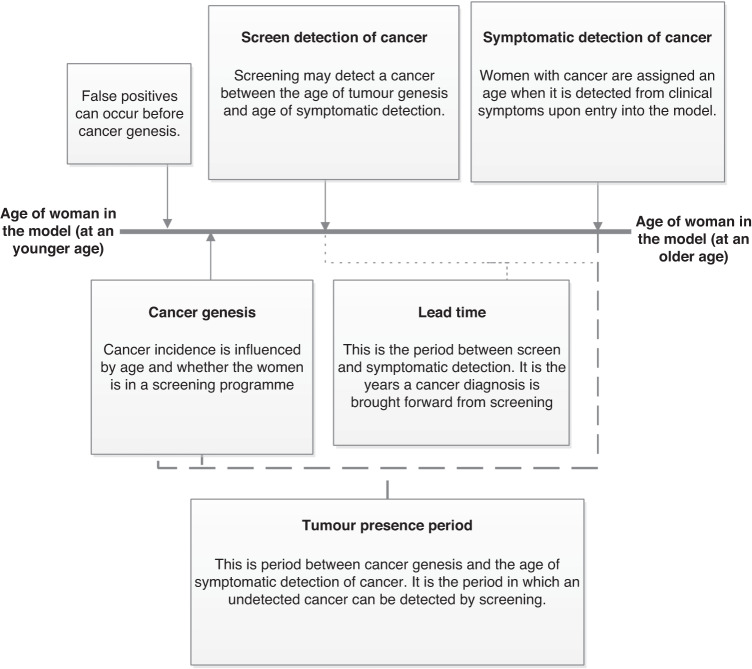
Natural history of cancer. Illustration of the progression of cancer in the model.

The ages for which a tumour can be screen-detected are established for woman with cancer before their first screening appointment, and do not change in their lifetime. First, the age a cancer is detected symptomatically is assigned. The duration of time a tumour grows (tumour presence period) before it is symptomatically detected is sampled from published age-based data with the mean duration varying from about 6 years (at age 35) to 8 years (at age 85) [22]. The ages of tumour visibility are the tumour presence period subtracted from the age at symptomatic detection. Screening can detect a tumour between the ages of tumour visibility and symptomatic detection, and screening FPs can occur outside this age range. Figure 2 illustrates the link between each aspect of this cancer history, and the impact of a screening upon cancer history.

### Model event: screening outcomes

In no screening, cancers are positively detected one they reach an age of symptomatic detection, which is set when women enter the model. If there is not an underlying tumour when a woman attends a screening appointment, there is chance of a FP result, which varies by the screening instrument used. A FP leads to a utility loss for the women and an increase in NHS costs due to further assessment of the screening result. If a woman has cancer, the chance of detecting a tumour is based on the sensitivity of the screening instrument. A positive test result leads to further assessment, which is assumed to confirm or refute the presence of the tumour. The sensitivity of digital mammography is influenced by both tumour size and breast percent density, and is estimated using a logistic function [22]. The maximum sensitivity of mammography is assumed to be 93%, which is the value observed in the UK screening of invasive tumours above 20 mm in diameter [23].

The size of a tumour is established by sampling size from tumour size distributions in the UK NHSBSP data at each screening appointment. First, a tumour is assigned to be Ductal carcinoma in situ (DCIS) or invasive based on age [24], and then its size is established based on tumour type (DCIS or invasive) tumours and age [25, 26]. For screening intervals of less than 3 years, this distribution is adjusted by the proportionate decrease in tumour sizes observed with more frequent screening in an UK RCT [27]. The final step is to adjust the sampled size to account for the size of cancers that are not detected at screening. This adjustment is based on the proportion of interval caners observed in the current NBCSP and on the size difference between screen and interval cancers observed in a recent national screening programme study [28]. A detailed explanation on the method used to establish the size of cancers, including summary tables of the tumour size distribution data that is applied, is presented in the Appendix (Section 4).

The diagnostic sensitivity of contrast-enhanced spectral mammogram, automated ultrasound and abbreviated MRI were introduced into the model by increasing the sensitivity of mammography by 47.37%, 54.55% and 61.54%, respectively. This was established based on the cancer detection rates of the instruments in the BRAID trial (personal communication with Fiona Gilbert, principal investigator on the BRAID trial) and the assumptions that the number of underlying number cancers is the same across trial arms (i.e., across detection instruments) and that all detected cancers were present (and missed) 18 months earlier at mammogram screening. ^.^The sensitivity of handheld ultrasound and full MRI is calculated by establishing the sensitivity of abbreviated MRI and abbreviated MRI and adjusting it to reflect the comparative performance of handheld ultrasound [29] and full MRI [30] from diagnostic studies. A summary of the screening sensitivity of instruments, FP rates and the sources used for diagnostic accuracy is in Table 2.

**Table 2:** Summary of the diagnostic accuracy of screening instruments and evidence sources.

Screening instrument	Sensitivity	Source and details of calculation	FP rate per screen	Source and details of calculation
Mammogram (MAM)	Screening sensitivity estimated using a logistic function. Maximum sensitivity assumed to be 93%.	logistic function [22] that depends on tumour diameter *d* (in mm), percent density m, and their interaction m/d^2^. The formulae is: *S*(*d*,*m*)= [exp(*β*1 + *β*2*d* +*β*3 *m* +*β*4 m/d^2^)] / [1 + exp(*β*1 + *β*2*d* + *β*3 *m* +*β*4 m/d^2^)] *β*1 = − 4.38 *β*2 = 0.49 *β*3 = − 1.34 *β*4 = − 7.18	Prevalent screen: 7.04% Incident screens: 2.23%	NHSBSP data [25]
Automated ultrasound	47.37% relative increase applied to the sensitivity of MAM	BRAID trial detection rate of cancers with automated ultrasound compared to MAM	Prevalent screen: 7.04% Incident screens: 2.23%	Assumed to be identical to MAM [45]
Handheld ultrasound	10.53% decrease applied to the sensitivity of AUS	Reflects the reduction in the comparative sensitivity of handheld ultrasound to automated ultrasound found in a sample of nearly 400 women [29]	Prevalent screen: 7.04% Incident screens: 2.23%	Assumed to be identical to MAM for handheld ultrasound alone [45] Hence, MAM combined with handheld ultrasound will have doubling of the false positive rate of MAM which is also found in a meta-analysis of studies [46]
Contrast-enhanced spectral mammogram	54.55% relative increase applied to the sensitivity of MAM	BRAID trial detection rate of cancers with contrast-enhanced spectral MAM compared to MAM	Prevalent screen: 7.14% Incident screens: 2.59%	contrast-enhanced spectral MAM has lower specificity compared to MAM in high breast density woman of 15.92% [47]
Abbreviated MRI	61.57% relative increase applied to the sensitivity of MAM	BRAID trial detection rate of cancers with abbreviated MRI compared to MAM	Prevalent screen: 7.32% Incident screens: 2.65%	Abbreviated MRI has lower specificity compared to MAM in high breast density woman of 18.82% [48]
Full MRI	2.22% relative increase applied to the sensitivity of AMRI	The pooled sensitivity for screening studies [30] was 0.90 for abbreviated MRI and 0.92 for full MRI	Prevalent screen: 7.60% Incident screens: 2.75% (for combined screening of MAM with MRI)	In the only study [49] that compared MAM to MAM with supplemental MRI the addition of MRI led specificity to increase by 23.31%

### Model event: interval between screening appointments, symptomatic detection of cancer

Between screening appointments, women will age, causing an age-related health loss. The only NHS costs incurred between screening appointments are the costs of inviting women to screening and to risk assessments. Women may or may not attend these appointments and, for women with cancers, there is a positive detection of cancer outside of the screening programme (at a GP appointment) if they reach their age of symptomatic detection. Between screening appointments, women can die from causes other than breast cancer. If this occurs in women with cancer, that cancer has gone undiagnosed over her lifetime and no cancer-related health losses or NHS treatment costs are incurred.

### Model events: initiation to risk assessment, invitation to screening appointment

Invitations to risk assessments are assumed to take place every 10 years. Age-specific data on the uptake to NHSBSP invitations stratified by screening history is used to calculate attendance to screen and risk assessment appointments [25]. These data are summarised in Appendix Table 2.

### Model event: cancer treatment and survival

Following the detection of a cancer, it is assigned to be either DCIS or an invasive TNM stage depending on age and mode of diagnosis using UK population screening data [24]. By adjusting for mode of dialysis, breast cancer-specific mortality is lower for screen-detected versus symptom-detected breast cancers for women with the same tumour stage at diagnosis. Several observational studies support this assumption [31, 32]. However, the absence of randomised controlled trials leaves room for debate regarding the independent impact of mode of detection on survival and therefore this assumption is tested in sensitivity analysis. The stage distribution is also adjusted by the duration of time from the women’s previously attended screening appointment to reflect the relative increases in the proportion of DCIS cancers and lower TNM stage cancers when screening intervals are shorter than 3 years for women, as observed in the United States (US) [33]. Further details are provided in the Appendix (Section 5).

At the time of detection, and each year after, women incur health-related losses due to treatment, and the NHS incurs treatment-related costs. Both are determined by the woman’s age at detection, the stage of cancer and the duration of time since detection of cancer. The treatment costs are based on evidence from observational studies of NHS costs incurred in the years after treatment. A detailed summary of all cost calculations, unit costs and sources are provided in Appendix Sections 6 (cancer treatment costs), 7 (screening and risk assessment costs) and 8 (health losses from cancer and from screening).

To establish an age of cancer death by DCIS, estimates from the English NHSBSP of annual excess risk by year since DCIS diagnosis [26] are applied to age-based ONS mortality statistics. For invasive cancers, the age-based ONS all-cause mortality probabilities from age of cancer detection is increased by survival hazards found to be statistically significant in a multivariable regression of UK women with breast cancer (covariates included: stage, age at cancer detection and mode of detection) [34]. The survival disadvantage for Stage 1 cancer is assumed to be the same as DCIS [35]. A summary of the invasive survival estimates is in the Appendix (Section 9).

### Economics

The population examined in the model is 20,000 women who enter the model at their first screening invitations (at ages 35–50 years). We assume that the only screening available to women receiving the current screening in the NBSCP is mammography at intervals of 3 years. Their health losses and costs incurred to the NHS are tracked over their lifetime until death (either by cancer or other causes), which can occur after national screening programmes have ceased to invite women to screening (at the ages of 70). A discount rate of 3.5% is used for both costs and health effects. Population health effects are measured by Quality Adjusted Life Years (QALYs). The QALY is a generic measure of disease burden. One QALY equates to 1 year in perfect health. The health and cost effects of RSBCS programmes were combined into a single outcome, the incremental cost-effectiveness ratio (ICER). This is defined as the incremental cost per incremental QALY gained when RSBCS are compared to current screening in the NHSBSP. As RSBCS are more costly than current screening in the NHSBSP, a positive ICER shows the average amount the NHS has spent on RSBCS to gain one QALY. New health technologies are typically considered to have produced a sufficiently large health gain in the population (“cost-effective”) when the ICER is below £20,000. NHS decision-makers do not routinely value a QALY above that price threshold. Results are also summarised via ‘net health benefit’ (NHB) per woman in the model’s population (20,000 women). This is how much the population health has improved in QALY terms and is a useful metric for policy because their goal is to maximising health in the population. NHB is the QALYs that the person being treated can expect minus the QALYs the health system loses owing to the opportunity costs with which their treatment is associated. For this, a value of £20,000 per QALY is used to present the opportunity costs of their treatment.

### Sensitivity and validity testing

Validity of the model is tested by comparing the model’s predicted NHSBSP outcomes to actual screening outcomes from the NHSBSP (tumour size and stage distribution of screen-detected cancers). The outcomes of the model is tested with probabilistic sensitivity analysis (PSA), and several deterministic sensitivity analyses that include altering the health losses from screening, the sensitivity of screening instruments, health losses from treatment and NHS treatment costs and removing the within TNM stage survival benefit from screen-detected cancers. The PSA method was an outer loop of 1000 standard Monte-Carlo draws. Probabilistic uncertainty is presented in a cost-effectiveness acceptability curve (CEAC) figure.

## Results

Table 3 shows the validation results. The results of the model’s precited cancer outcome for the current screening programme closely matches the tumour characteristics (size and stage) observed in NHSBSP data. There is a slight overprediction of Stage 4 cancers in the model (0.85% in the model results compared to the rate of 0.091% in the audit data). This will slightly improve the cost-effectiveness of risk-stratified regimens when compared to the current screening programme due to there being a greater number of advanced-stage tumours that risk-stratified screening can detect earlier. Conversely, it will diminish the cost-effectiveness of the current screening programme when compared to having no screening programme in place.

**Table 3:** Validation results, NBCSP screening outcomes compared to the model prediction of NBCSP screening outcomes.

Description of validation target outcomes	NBCSP screening outcomes (target outcomes)	Model prediction	Reference for target outcomes
Percentage of tumours that are DCIS at screen detection	21.1%	21.58%	Data request to the National Audit of Breast Cancer in Older Patients [24]
Invasive cancer TNM stage distribution for cancers detected by screening. Percentage in each stage			
Stage 1	66.00%	66.97%	
Stage 2	28.89%	27.84%	
Stage 3	4.19%	4.34%	
Stage 4	0.091%	0.85%	
Size of cancers detected at screening			
Less than 20 mm (DCIS)	62.76%	55.54%	Table 1 in Mannu et al. [26]
Above 20 mm (DCIS)	37.24%	44.46%	
Less than 20 mm (Invasive)	66.00%	56.02%	Table 2 in Breast Screening Frequency Trial Group, 2002 [27]
Above 20 mm (Invasive)	34.00%	43.98%	

Table 4 shows the base-case results of all scenarios considered in the model. Compared with the current screening programme, RSBCS programmes generated additional costs and additional QALYs and the current screening programme also generated more costs and QALYs than no screening. PROCAS brought greater net health benefit than the NHSBSP, no screening programme and all other RSBCS programmes. This conclusion may also be observed by considering the NHB values, which show the number of the QALYs gained in the population (per woman invited to screening) after accounting for the QALYs lost owing to the money the NHS has spent on additional screening (that could have been spent elsewhere to improve health). The NHB were largest for PROCAS at 16.611 QALYs per woman invited for screening. ASSURE 1 provided the second largest NHB of 16.591. This means choosing PROCAS instead of ASSURE would provide the NHS a net amount of 0.02 QALYs per woman invited to screening. This same conclusion is also expressed from the ICER results (Online Appendix Section 10). Hence, PROCAS is associated with the greatest net benefit because health is gained at a cheaper cost to the NHS than if ASSURE is used. PROCAS also obtains more QALYs from fewer screens per woman compared to the BRAID regimens. All risk-stratified screening regimens had a larger percentage of tumours being diagnosed at an earlier stage in comparison to both the current screening programme and the absence of screening. It is important to highlight that PROCAS, with its starting age of 35 instead of 50, results in a higher total number of diagnosed tumours. This simply indicates that some women develop cancer before reaching the age of 50. The ICER for the NHSBSP compared with no screening is below £20,000 (£12,522). This suggests decision-makers are correct to have a screening programme, because the NBCSP brings a net health benefit compared to no population screening.

**Table 4:** Base case economic results of RSBCS programmes.

Regimen	Undiscounted values per women			Discounted values per women			No. of screens per women	Total Diagnosis per 20,000 women		
	Total cost	Total QALYs	Life years	Disc. costs	Disc. QALYs	NHB		DCIS	Inv.	Stage 3–4
No screening	£2411	27.931	35.809	£1188	16.613	16.554	0.00	124	2613	580
PROCAS (PROCAS with a starting age of 50)	£3323 (£2915)	28.180 (28.126)	36.065 (36.020)	£1971(£1743)	16.710(16.693)	16.611 (16.605)	6.30(5.24)	376 (324)	2827 (2412)	491 (403)
ASSURE 1	£2805	28.089	36.002	£1641	16.673	16.592	5.50	280	2457	414
ASSURE 2	£2821	28.077	35.995	£1649	16.667	16.585	5.94	294	2442	425
ASSURE 3	£2953	28.040	35.981	£1743	16.648	16.560	4.54	244	2492	433
ASSURE 4	£3059	28.073	36.005	£1825	16.663	16.572	5.23	296	2441	422
BRAID 2	£3093	28.099	36.019	£1876	16.676	16.582	8.49	315	2422	407
BRAID 3	£3000	28.092	36.022	£1804	16.671	16.581	8.49	321	2415	415
BRAID 4	£3229	28.100	36.024	£1972	16.676	16.577	8.49	317	2420	406
Current screening	£2703	28.023	35.963	£1537	16.641	16.564	4.68	252	2485	469

*BRAID* Breast Screening Risk Adaptive Imaging for Density, *ASSURE* Adapting Breast Cancer Screening Strategy Using Personalised Risk Estimation, *PROCAS* Predicting Risk of Cancer at Screening.

Results for all scenario analysis, PSA and CEAC figure are provided in the Appendix (Online Appendix Section 10). Cost-effectiveness results for scenario analysis, including removing the within stage survival advantage of screen-detected cancer (Appendix Table 23), and for the PSA are very similar to the base case in that PROCAS providing the most NHBs followed by ASSURE 1. No screening, current screening, and ASSURE 3 always generate the least NHB. The CEAC indicates that at a threshold of between £20,000 and £30,000 there exists less than 1% probability of current practice being optimal, with all other risk-stratified screening interventions having a higher probability. The CEAC curves for the risk-stratified screening interventions are close together, suggesting that the model lacks a high level of confidence in identifying the optimal approach among many of the different risk-stratified screening regimens.

## Discussion

There is national and international interest in whether introducing new risk-stratification approaches that are linked to RSBCS programmes into national screening programmes will improve the overall health of the population. The problem facing health policy decision-makers is that this can not be known from available empirical research alone. For instance, evidence on the effect of risk stratification comes from sub-populations of the screened women population and typically from evidence that observes screened women for only a few years. These problems are overcome in this study by combining existing evidence in an economic model to make a prediction of the effect of RSBCS programmes for the entire population of adult women in the UK from their first screen over their lifetime. The economic model predicts outcomes from three forms of RSBCS (ASSURE, PROCAS and BRAID) in comparison to no screening and the current screening in the NHSBSP. There are other potential models for RSBCS, which will be a target for future research [36].

The results show that the NHSBSP is an effective use of NHS resources compared to no screening programme and all three RSBCS regimens extend life expectancy compared to current screening. PROCAS is the most expensive approach, but it also brought the most population net health benefits, and the health gains were sufficient in magnitude to justify additional screening costs when compared with the next-best option (ASSURE 1). PROCAS is a regimen that requires a baseline mammogram, a risk assessment survey (TC instrument) and genetic test. It removes screening in low-risk woman and offers medium and high-risk woman more frequent screening; biennial and annual, respectively.

The ASSURE and BRAID regimens offer more frequent screening than the NHSBSP and bring a net health benefit despite the very different screening programmes on offer. This is evidence that the benefits of screening outweigh its harms. This remained the case even in the scenario analysis where the benefits of screening were reduced by lowering the diagnostic accuracy of all screening instruments by 10%, and its harms were increased by doubling the disutility losses from screening.

A recent systematic review of international RSBCS economic models reported that nine of the ten reviewed studies [37] found risk-stratification improved population health and were a worthwhile use of resources after accounting for the additional healthcare spending required by RSBCS programmes. PROCAS and BRAID have not previously been evaluated, and ASSURE 1 been found to be an effective policy option in the only previous study that evaluated its health and cost consequences on UK adult women [38]. Therefore, the findings reported here are in agreement with the existing literature.

A strength of this evaluation is that it is the first model to compare a range of programmes that vary by the cancer screening instruments used, starting ages for screening, frequency of screening, number of risk groups, and thresholds that define risk grouping. This is also the first model of national screening that examines different instruments to assess risk and replicates the intended way RSBCS is intended to be introduced, where some women move into different risk groups as they age (reflecting a change in their risk of cancer incidence), and in doing so are offered a new screening programme.

The model has some limitations. While the model is based on UK sources there are a small number of exceptions. The mammogram sensitivity function (that is based on tumour size and breast density), and tumour presence times are derived from the Swedish NHSBSP. The increase in the rate of overdiagnosis and shift to less advanced tumours at detection when screening intervals are shorter than in the UK NHSBSP are taken from national population screening programmes in European and US women, respectively. In the model, cancer stage is assigned independently of tumour size at a screening appointment while in clinical practice tumour size is a prognostic factor used to classify cancer stages. The breast cancer treatment costs were taken from papers that reflect care from 2001 to 2010 [39] and 2014 to 2016 [40]. Despite being uplifted to account for inflation, over 20 new breast cancer treatments have been approved by NICE for use in the NHS since the beginning of 2016, which may mean that the costs used in the model are an underestimate.

The model assumes that breast cancer detected by screening is an independent prognostic factor in breast cancer mortality (independent of diagnosis at younger age and at an earlier stage) which causes a substantially more favourable survival rate. This is controversial because the reason why screening should be independent prognostic factor survival is not well-understood and therefore drawing a credible quantitative conclusion as to the extent of survival advantage is not easy. However, it has been the focus of much research and the survival estimate we use [32] is similar to that reported by others who have shown the method of detection to be an independent prognostic factor [41–44]. Without this assumption in the model, PROCAS still remained an effective use of NHS resources compared to other risk-stratified screening regimens and to current screening,

The model does not consider the budget or capacity implications of alternative screening regimens. The PROCAS regimen has the potential to cause capacity bottlenecks because the NHS would be required to increase the number of screens women receive. The model found women were screened on average 4.7 times in the current screening programme compared to 6.3 with PROCAS. Without a corresponding reduction in screening elsewhere in the system, or expansion of screening capacity, this could impact on waiting times for breast screening appointments. Scaling up the national breast cancer screening programme to provide a greater number of annual screens may increase short-term costs from inefficiencies associated with the process of switching from a decades old approach of age-based mammography to a mix of programmes in a population segmented by risk. Hence, further research is needed to consider the long-term budget impact of RSBCS population-based screening programmes.

In conclusion, this study shows that risk-based screening is more beneficial for women at the population level than the UK NHSBSP, which itself is an effective use of NHS resources compared to no screening programme. However, all forms of RSBCS are more resource-intensive. Given the large scale of screening in the NHSBSP, future research should consider the feasibility and capacity implications of introducing RSBCS.

### Supplementary information

Online Appendix

## Data Availability

No data were collected for this study. All data used in the economic model are taken from the research literature and referenced in the manuscript.
