# Supplementary material for: The cost-effectiveness of risk-stratified breast cancer screening in the UK
Source: Br J Cancer. 2023 Oct 17;129(11):1801–9. doi: 10.1038/s41416-023-02461-1 (PMC10667489; doi:10.1038/s41416-023-02461-1)
Supplement: Supplementary file 1 — Online Appendix [file 41416_2023_2461_MOESM1_ESM.docx]

**Appendix Summary:** This appendix contains the parameter information and sources used as inputs into the economic model (Section 1 to Section 9) and sensitivity analysis of model results (Section 10).

**Section 1 – key model parameters**

Appendix Table 1: Estimating the incidence in the absence of screening

| Female breast cancer incidence per 100,000 by age, observed and estimated for the scenario with no screening programme. | | | |
| --- | --- | --- | --- |
| Year of age | Observed incidence from ONS | Incidence without screening | |
|  |  | Unadjusted | Adjusted for self-selection bias |
| 50 | 273.6 | 236.1 | 244.8 |
| 51 | 273.6 | 236.1 | 244.8 |
| 52 | 273.6 | 236.1 | 244.8 |
| 53 | 273.6 | 235.1 | 243.7 |
| 54 | 273.6 | 235.1 | 243.7 |
| 55 | 270.0 | 235.1 | 243.7 |
| 56 | 270.0 | 233.0 | 241.6 |
| 57 | 270.0 | 233.0 | 241.6 |
| 58 | 270.0 | 233.0 | 241.6 |
| 59 | 270.0 | 284.1 | 294.4 |
| 60 | 343.9 | 284.1 | 294.4 |
| 61 | 343.9 | 284.1 | 294.4 |
| 62 | 343.9 | 298.0 | 308.9 |
| 63 | 343.9 | 298.0 | 308.9 |
| 64 | 343.9 | 298.0 | 308.9 |
| 65 | 399.9 | 360.4 | 373.4 |
| 66 | 399.9 | 360.4 | 373.4 |
| 67 | 399.9 | 360.4 | 373.4 |
| 68 | 399.9 | 390.0 | 403.0 |
| 69 | 399.9 | 390.0 | 403.0 |
| 70 | 330.1 | 390.0 | 403.0 |
| 71 | 330.1 | 390.0 | 403.0 |
| 72 | 330.1 | 390.0 | 403.0 |
| 73 | 330.1 | 390.0 | 403.0 |
| 74 | 330.1 | 390.0 | 403.0 |
| 75 | 379.6 | 390.0 | 403.0 |
| 76 | 379.6 | 390.0 | 403.0 |
| 77 | 379.6 | 390.0 | 403.0 |

The probability of adult women having cancer at any given age is taken from ONS lifetable statistics^12^. At ages between 50 and 77 we lower this by age-based excess percentage incidence in Appendix Table 1, which is the age-based percentage difference between the observed incidence from ONS and the (lower) estimates of incidence without screening after adjustment for self-selection bias^14^. This gives the probability of having cancer under no screening at ages between 50 and 77 under no screening. We assume women below the age of 50 have the excess percentage difference of a 50 year old, and women above 77 years have the excess percentage difference of a 77 year old. This probability distribution is converted to cumulative risk to give the lifetime probability of cancer given a particular age. This is done by assuming a constant risk within the year. The model samples from the cumulative risk distribution to establish if a woman has cancer under the no screening scenario.

Appendix Table 2: Probability of attending a screening appointment

| **Age-group (years)** | **First invite** | **Subsequent invite:** | |
| --- | --- | --- | --- |
|  |  | **Previous non-attender** | **Previous attender** |
| **Less than 45** | 66.5% | 33.0% | 82.8% |
| **45 to 49** | 66.5% | 33.0% | 82.8% |
| **50 to 52** | 67.4% | 34.3% | 84.1% |
| **53 to 54** | 37.7% | 27.5% | 85.3% |
| **55 to 59** | 31.9% | 17.5% | 86.1% |
| **60 to 64** | 29.8% | 10.3% | 87.5% |
| **65 to 70** | 26.9% | 7.0% | 88.4% |
| **Over 70** | 27.0% | 5.9% | 84.2% |

Appendix Table 3: Distribution of cumulus breast density by age of entry into the model

| Age of entry into the model (years) | Cumulus percent density* | | | | | |
| --- | --- | --- | --- | --- | --- | --- |
|  | **0%** | **1-10%** | **11-24%** | **25-49%** | **50-74%** | **75%+** |
| 60 to 71 | 13.13% | 15.66% | 18.69% | 29.80% | 15.15% | 7.58% |
| 55 to 59 | 11.57% | 17.16% | 14.93% | 22.39% | 20.52% | 13.43% |
| 49 to 54 | 5.56% | 8.64% | 14.81% | 22.84% | 25.31 | 22.84% |
| 45 to 48 | 2.31% | | | 20.61% | 71.44% | 5.64% |
| 40 to 44 | 1.25% | | | 17.61% | 71.32% | 9.82% |
| 35 to 39 | 1.57% | | | 17.22% | 69.47% | 11.74% |
| 30 to 34 | 1.91% | | | 20.23% | 16.41% | 61.45% |
| 25 to 29 | 0.00% | | | 26.61% | 14.68% | 58.72% |
| 19 to 24 | 0.00% | | | 46.15% | 15.38% | 38.46% |

* After assigning women to a cumulus breast density category, women were given a specific cumulus breast density value by assuming a uniform distribution of risk within the category

Appendix Table 4: Distribution of risk threshold categories.

| **Risk threshold category (%)*** | **With genetic component (PROCAS)** | | **Without genetics (ASSURE)** | |  |
| --- | --- | --- | --- | --- | --- |
|  | **No cancer** | **Cancer** | **No cancer** | **Cancer** | |
| 0 to 1 (no genetics) 0 to 1.49 (genetics) | 17% | 4% | 2% | 5% | |
| 1.50 to 1.99 | 15% | 2% | 28% | 10% | |
| 2.00 to 3.49 | 33% | 11% | 42% | 13% | |
| 3.50 to 4.99 | 17% | 16% | 16% | 21% | |
| 5.00 to 7.99 | 12% | 21% | 9% | 24% | |
| 8.00 to 12.00 | 6% | 46% | 3% | 27% | |

* After assigning women to a Tyrer-Cuzick ten year risk threshold category, women were given a specific risk score by assuming a uniform distribution of risk within the category

Appendix Table 5: Annual decline in cumulus percent density by age and density

| Current age in the model | Annual decline in cumulus percent density | | |
| --- | --- | --- | --- |
|  | Cumulus percent density at the start of the year is less than 25% | Cumulus percent density at the start of the year is between 25% and 75% | Cumulus percent density at the start of the year is greater than 75% |
| 56 or under | 1.73 | 2.27 | 1.2 |
| Between 57 and 61 | 2.2 | 1.8 | 1 |
| Between 62 and 66 | 1.4 | 0.8 | 1.6 |
| Between 67 and 74 | 1.2 | 0.8 | 1 |
| 75 and over | 0 | 0 | 0 |

**Section 2: establishing the consequences of overdiagnosis**

Overdiagnosis in a breast cancer screening program is the diagnosis, as a result of screening, of cancer that would not have been made in the women’s lifetime had she not been screened. We reduce cancer incidence by the rate of overdiagnosis (3.7%) in the no screening scenario compared to the current screening programme, as this is the most recent excess cancer estimate for the NHSBSP^14^. Cancer incidence rate for scenarios of three yearly screening in the model (such as the current NHSBSP) is not altered, it is the rate is the current breast cancer incidence rate in the UK.

The overdiagnosis rate must increase when screening is more frequent than the current screening programme. To account for this, at cancer detection the model examines if the woman was receiving a 3 yearly, two yearly or one yearly screening programme. If the woman was receiving more frequent screening than 3 yearly her costs and QALYS accrued from cancer detection is increased by the percentage increase in cancer incidence (taken from the literature) of two or one yearly screening compared to three yearly screening. Hence, this approach applies QALYs and costs weights (with the weights being the increase in overdiagnosis from more frequent screening than 3 years), rather than increasing the sampled cancer incidence. This approach fully reflects the effect of increased cancer incidence under more frequent screening than three years via an increase in woman’s costs and QALYS accrued from cancer.

The use of weights is required because cancer incidence is sampled upon entry into the model. If overdiagnosis is introduced by altering the incidence rate the effect of overdiagnosis is constrained be the constant over a woman’s lifetime. Changing cancer incidence works when accounting for overdiagnosis in the current UK screening programme compared to no screening because there is no change in screening frequency in the current UK screening programme. However, it can not work if women move between different screening over their lifetime. This is because the screening programmes women receive over their lifetime are established from running the simulation, hence the programmes are not known at the moment the model samples cancer incidence (upon a woman’s entry into the model and prior to their first screen).

The sources for the weights are as follows. It was assumed that the overdiagnosis rate for two yearly screening compared to no screening was 3.85% which is the mean overdiagnosis rate for EU countries with 2-yearly routine screening as reported in a literature review on overdiagnosis in mammographic screening for breast cancer in Europe^16^. This was further assumed to represent a relative 4.05% increase (i.e., 3.85/3.7) in the incidence of cancer moving from screening every three years to two years. If the duration of time between screen detection of cancer and the previous attendance at a screening appointment was between 18 months and 30 months, the overdiagnosis rate for a two-yearly screening programme was applied. Upon screen detection of cancer, both the subsequent cancer treatment costs and the subsequent QALY losses accrued over a woman’s lifetime are increased by 4.05%. In absence of any other evidence for the overdiagnosis rate for annual screening, the same relative change was assumed (a 4.05% increase) when moving from two-yearly screening to one-yearly screening.

**Section 3 - Risk assessments (TC score for PROCAS and ASSURE regimens)**Assigning risk scores conditional on cancer incidence means that the accuracy of the risk assessment tools, investigated in two published observational studies from the PROCAS project^6,20^, are accounted for in the model. When assigning risk scores for women with cancer, the distribution of the risk scores of 57,902 women aged between 46 and 73 years from the Greater Manchester area (recruited in the PROCAS trial) is assumed to be representative of population risk distribution in the UK. The 10-year Tyrer-Cuzick for women without cancer was sampled from the probability distribution for women at baseline in the PROCAS study because at baseline none of the women had cancer. For women with cancer, the 10-year risk score in the PROCAS study corresponds to an assessment taken in their last risk assessment prior to the date cancer is detected. The age of that risk assessment is reproduced in the model by subtracting from the woman’s age of cancer diagnosis (assumed to be their age of symptomatic cancer detection) the mean follow up period in PROCAS, which is the duration from risk assessment to cancer diagnosis. The 10-year risk score at all other ages is extrapolated from this base risk using the cross-sectional relationship between UK adult women’s age and 10-year Tyrer-Cuzick risk, which included breast density as a risk factor. Consequently, the change in risk from one year to the next varies in the model resulting in smaller increases in 10-year risk in the years between 50 and 60 than between 60 and 70 years of age. The annual change in 10-year Tyrer-Cuzick risk is assumed to be identical for both types of Tyrer-Cuzick assessment methods (whether augmented by breast density alone or by breast density and genetic factors), as genetic factors do not change over time.

**Section 4 - cancer sizes for undetected cancers at screening appointment**

The sampling of tumour sizes takes the following approach. Based on age and duration of time between her current and previous screening appointment, the tumour is temporarily classified as DCIS or invasive based on age^24^. If invasive, a size category (less than 10mm, equal to or greater than 10mm and less than 20, equal to or greater than 20mm and less than 50 or equal to or greater than 50mm) is assigned from a probability density distribution of age-based invasive cancer tumour sizes. These data are taken data from breast cancer national screening programme data in England^25^. The size distribution shows larger tumours for younger women which reflects the real-world situation of faster growing tumours in younger women. If the tumour is assigned to be DCIS, the size distribution is taken from data from over 35,000 women in England in the current screening programme, and the same tumour size categories as invasive cancers is applied^26^. Both size distributions (DCIS and invasive) are adjusted for the decrease in tumour sizes observed with annual screening in the only UK RCT exploring the impact of shorter screening intervals compared to the current approach of three yearly screening^27^. Extrapolation for screening less frequent than 3 years is based on an assumed constant linear change in the percentage of tumours detected in different size categories observed in 3 yearly screening when moving through less frequent screening regimens to one yearly screening. A summary table describing showing the size modifiers, and how they were derived is presented in Appendix Table 6.

Appendix Table 6: Tumour size estimates for screening intervals of less than three years

| Tumour size categories (mm) | Percentage observed under 1 yearly screening* | estimated percentage observed under 1.5 yearly screening | estimated percentage observed under 2 yearly screening* | Percentage observed under 3 yearly screening* | Percent difference in 3 yearly screening compared to 1 yearly | Percent difference in 3 yearly screening compared to estimated 1.5 yearly screening | Percent difference in 3 yearly screening compared to estimated 2 yearly screening |
| --- | --- | --- | --- | --- | --- | --- | --- |
| less than 10 | 25% | 23.5% | 22% | 19% | 6% | 4.5% | 3% |
| Equal to or greater than 10mm and less than 20 | 48% | 47.75% | 47.5% | 47% | 1% | 0.75% | 0.5% |
| Equal to or greater than 20mm and less than 50 | 25% | 26.75% | 28.5% | 32% | - 7% | - 5.25% | - 3.50 |
| Equal to or greater than 50mm | 2% | 2% | 2% | 2% | No change | No change | No change |

*data taken from Table 2 in Breast Screening Frequency Trial Group (2002)^27^.

The resulting tumour size density distributions by age and screening frequency is presented in Appendix Table 7 and Appendix Table 8 for DCIS and invasive tumours respectively. In brief, the tumour size modifiers derived in Appendix Table 3 (the three right most columns) and reproduced in Appendix Table 7 (the three right most columns). The same size modifies are applied to the DCIS size distribution under the current NHSBSP (the fifth column to the left in Appendix Table 7) to predict the tumour size distribution under more frequent screening (the second, third and fourth columns in Appendix Table 7). For invasive cancers the same approach is followed. The same tumour size modifiers are applied to the tumour size distribution for invasive cancers under three yearly screening (the first six rows of Table 8) to establish the size destitutions under more frequent screening regimens (row 8 and all rows below it in Table 8).

Appendix Table 7: Probability density distribution of DCIS tumour size by time since last screening appointment

| Size of tumour (mm) | Estimated percentage observed under: | | | | Percent difference in 3 yearly screening compared to | | |
| --- | --- | --- | --- | --- | --- | --- | --- |
|  | 1 yearly screening* | 1.5 yearly screening | 2 yearly screening* | 3 yearly screening | 1 yearly | 1.5 yearly screening | 2 yearly screening |
| < 10 | 38.90% | 37.40% | 35.90% | 32.90% | 6% | 4.5% | 3% |
| 10 to < 20 | 30.86% | 30.61% | 30.36% | 29.86% | 1% | 0.75% | 0.5% |
| 20 to < 50 | 22.69% | 24.44% | 26.19% | 29.69% | - 7% | - 5.25% | - 3.50 |
| ≥ 50 | 7.55% | 7.55% | 7.55% | 7.55% | No change | | |

* size distribution has been estimated by applying the estimates in the right columns, of the change in distribution when compared 3 yearly screening (established in Appendix Table 6); these estimates are applied to the cancer size distribution by screening frequency. DCIS size data under the current screening programme (3-yearly screening) is taken from Table 1 in Mannu *et al* 2020^26^

Appendix Table 8: Probability density distribution of invasive tumour size by age and time since last screening appointment

| Duration from last screening appointment (months)* | Screening regimen from which probabilities are derived | Age bands | Tumour size < 10mm | 10mm ≤ tumour size < 20mm | 20mm ≤ tumour size < 50mm | Tumour size ≥ 50mm |
| --- | --- | --- | --- | --- | --- | --- |
| ≥ 30 | Three-yearly | ≤49 | 24.86% | 42.69% | 29.03% | 3.42% |
| ≥ 30 | Three-yearly | 50-54 | 22.82% | 45.90% | 26.98% | 4.30% |
| ≥ 30 | Three-yearly | 55-59 | 25.32% | 46.93% | 24.39% | 3.35% |
| ≥ 30 | Three-yearly | 60-64 | 27.03% | 45.60% | 24.66% | 2.71% |
| ≥ 30 | Three-yearly | 65-70 | 27.38% | 46.89% | 23.24% | 2.49% |
| ≥ 30 | Three-yearly | ≥70 | 26.87% | 45.98% | 24.45% | 2.70% |
| 18 to 30 | Two-yearly | ≤49 | 27.86% | 43.19% | 25.53% | 3.42% |
| 18 to 30 | Two-yearly | 50-54 | 25.82% | 46.40% | 23.48% | 4.30% |
| 18 to 30 | Two-yearly | 55-59 | 28.32% | 47.43% | 20.89% | 3.35% |
| 18 to 30 | Two-yearly | 60-64 | 30.03% | 46.10% | 21.16% | 2.71% |
| 18 to 30 | Two-yearly | 65-70 | 30.38% | 47.39% | 19.74% | 2.49% |
| 18 to 30 | Two-yearly | ≥70 | 29.87% | 46.48% | 20.95% | 2.70% |
| 12 to 18 | 18-monthly | ≤49 | 29.36% | 43.44% | 23.78% | 3.42% |
| 12 to 18 | 18-monthly | 50-54 | 27.32% | 46.65% | 21.73% | 4.30% |
| 12 to 18 | 18-monthly | 55-59 | 29.82% | 47.68% | 19.14% | 3.35% |
| 12 to 18 | 18-monthly | 60-64 | 31.53% | 46.35% | 19.41% | 2.71% |
| 12 to 18 | 18-monthly | 65-70 | 31.88% | 47.64% | 17.99% | 2.49% |
| 12 to 18 | 18-monthly | ≥70 | 31.37% | 46.73% | 19.20% | 2.70% |
| ≤ 12 | Annual | ≤49 | 30.86% | 43.69% | 22.03% | 3.42% |
| ≤ 12 | Annual | 50-54 | 28.82% | 46.90% | 19.98% | 4.30% |
| ≤ 12 | Annual | 55-59 | 31.32% | 47.93% | 17.39% | 3.35% |
| ≤ 12 | Annual | 60-64 | 33.03% | 46.60% | 17.66% | 2.71% |
| ≤ 12 | Annual | 65-70 | 33.38% | 47.89% | 16.24% | 2.49% |
| ≤ 12 | Annual | ≥70 | 32.87% | 46.98% | 17.45% | 2.70% |

The above tumour size distributions are based on data from screen detected cancers. However, not all cancers are present at a screening appointment are detected at screening. Therefore, the tumour size distribution is adjusted to include the sizes of cancers that are not detected and present symptomatically after an appointment (interval cancers). The probability that the cancer is an interval cancer is based on a study that examines the number of interval cancers observed in the UK breast cancer service screening program^28^. The probability varies by age, duration of time between the current and previous screening appointment, and if the screening appointment was a woman’s first or not. The probability decreases as the screening interval shortens, for older women and for the first screen. Values are presented in Appendix Table 9.

Appendix Table 9: Probability density distribution of interval cancer by age and time since last screening appointment

| Duration from last screening appointment (months) | Age bands | Probability of interval cancer: | |
| --- | --- | --- | --- |
|  |  | First screen | Subsequent screens |
| Above 24 to 36 | 54 and under | 27.03% | 35.92% |
| Above 24 to 36 | 55-59 | 25.12% | 31.87% |
| Above 24 to 36 | 60-64 | 26.39% | 27.28% |
| Above 24 to 36 | 65-69 | 13.74% | 23.51% |
| Above 24 to 36 | 70 and over | 16.00% | 18.56% |
| Above 12 to 24 | 54 and under | 17.61% | 23.83% |
| Above 12 to 24 | 55-59 | 16.13% | 21.02% |
| Above 12 to 24 | 60-64 | 16.78% | 17.21% |
| Above 12 to 24 | 65-69 | 8.99% | 14.17% |
| Above 12 to 24 | 70 and over | 8.70% | 10.56% |
| 12 or less | 54 and under | 6.36% | 9.01% |
| 12 or less | 55-59 | 5.11% | 7.59% |
| 12 or less | 60-64 | 6.54% | 5.56% |
| 12 or less | 65-69 | 4.27% | 4.68% |
| 12 or less | 70 and over | 4.55% | 3.41% |

The positive relationship of interval cancers with younger ages and longer screening intervals have also been confirmed in screening programmes outside of the UK^50,51^. The probabilities are established by assuming that rate of interval cancers in the UK breast cancer service screening program^28^ consists entirely of cancers that were present at their last screening appointment but were not detected, thereby leading to symptomatic cancer detection. Thus, we assume none of the interval cancers in the data could have had their genesis after the women’s last screening appointment and have advanced in stage to where the cancer is detected symptomatically in a period under three years (i.e., in a period before her next scheduled screening appointment). This assumption is plausible given the tumour presence times of cancers, as described in the main text, has been established to be a minimum of six years.

The size of interval cancers is established by first sampling from the tumour size distribution of screen detected cancers, and adjusting this by the size difference between screen and interval cancers observed in a recent national screening programme study^52^. The size adjustment is based on the breast density of the woman. For women with VG1 (0% to 4.5% Volpara density), VG2 (4.6% to 7.5%), VG3 (7.6% to 15.5%) and VG4 (>15.5%) the sampled size is increased by 9mm, 6mm, 7mm and 4mm respectively. This represents the well-established relationship that cancers in women with dense breasts tend to be larger at detection which may be due to more rapidly growing tumours, delayed detection, or both^53^. The resulting distribution of interval cancers is presented in Appendix Table 10.

Appendix Table 10: Probability density distribution of interval cancer by age and time since last screening appointment

| Duration from last screening appointment (months)* | Screening regimen from which probabilities are derived | Age bands | Tumour size < 10mm | 10mm ≤ tumour size < 20mm | 20mm ≤ tumour size < 50mm | Tumour size ≥ 50mm |
| --- | --- | --- | --- | --- | --- | --- |
| ≥ 30 | Three-yearly | ≤49 | 24.86% | 42.69% | 29.03% | 3.42% |
| ≥ 30 | Three-yearly | 50-54 | 22.82% | 45.90% | 26.98% | 4.30% |
| ≥ 30 | Three-yearly | 55-59 | 25.32% | 46.93% | 24.39% | 3.35% |
| ≥ 30 | Three-yearly | 60-64 | 27.03% | 45.60% | 24.66% | 2.71% |
| ≥ 30 | Three-yearly | 65-70 | 27.38% | 46.89% | 23.24% | 2.49% |
| ≥ 30 | Three-yearly | ≥70 | 26.87% | 45.98% | 24.45% | 2.70% |
| 18 to 30 | Two-yearly | ≤49 | 27.86% | 43.19% | 25.53% | 3.42% |
| 18 to 30 | Two-yearly | 50-54 | 25.82% | 46.40% | 23.48% | 4.30% |
| 18 to 30 | Two-yearly | 55-59 | 28.32% | 47.43% | 20.89% | 3.35% |
| 18 to 30 | Two-yearly | 60-64 | 30.03% | 46.10% | 21.16% | 2.71% |
| 18 to 30 | Two-yearly | 65-70 | 30.38% | 47.39% | 19.74% | 2.49% |
| 18 to 30 | Two-yearly | ≥70 | 29.87% | 46.48% | 20.95% | 2.70% |
| 12 to 18 | 18-monthly | ≤49 | 29.36% | 43.44% | 23.78% | 3.42% |
| 12 to 18 | 18-monthly | 50-54 | 27.32% | 46.65% | 21.73% | 4.30% |
| 12 to 18 | 18-monthly | 55-59 | 29.82% | 47.68% | 19.14% | 3.35% |
| 12 to 18 | 18-monthly | 60-64 | 31.53% | 46.35% | 19.41% | 2.71% |
| 12 to 18 | 18-monthly | 65-70 | 31.88% | 47.64% | 17.99% | 2.49% |
| 12 to 18 | 18-monthly | ≥70 | 31.37% | 46.73% | 19.20% | 2.70% |
| ≤ 12 | Annual | ≤49 | 30.86% | 43.69% | 22.03% | 3.42% |
| ≤ 12 | Annual | 50-54 | 28.82% | 46.90% | 19.98% | 4.30% |
| ≤ 12 | Annual | 55-59 | 31.32% | 47.93% | 17.39% | 3.35% |
| ≤ 12 | Annual | 60-64 | 33.03% | 46.60% | 17.66% | 2.71% |
| ≤ 12 | Annual | 65-70 | 33.38% | 47.89% | 16.24% | 2.49% |
| ≤ 12 | Annual | ≥70 | 32.87% | 46.98% | 17.45% | 2.70% |

In summary, the application of probability density distributions for screen and interval cancer by age and time since last screening appointment is to establish the size of an undetected cancer at a screening appointment using data from the UK screening programme. The outcome is that the size of an undetected tumour immediately prior to screening will be smaller for younger women and with less frequent screening. Such relationships with interval cancers are well-established in the research literature^50, 51^.

Cancers that are undetected at a screening appointment are assigned a cancer size at subsequent screening appointments independently of its previously assigned size. Given that the woman will be older at her next screening appointment and that cancer size is assigned conditional on age, then at the population level the size of undetected cancers is larger at subsequent appointments. On average, this will also be the case for individual woman in the model simulation. In this manner, the model represents growth in the size of undetected cancers between screening appointments. However, for individual woman in the model simulation, the process of sampling cancer sizes at each screening appointment can lead an undetected cancer to reduce in size at a subsequent screening appointment. This may be a merit of the model as in some cases this has been observed in the natural history of breast cancer: breast cancer tumours, even those that grow to a size where they can be detected on mammograms, can stop growing, shrink and disappear often over a period of less than two years.

**Section 5 – Establishing a cancer stage when a cancer is detected**

A key benefit of cancer screening is the effect it has on the cancer stage of detected cancers. This effect is accounted for in the model in three ways: (1) screening means the cancer is detected at an earlier age (i.e. at an age earlier than the age of symptomatic detection) and younger woman are less likely to have advanced cancer for any method of detection. (2) screen detected cancers are less likely to be advanced than those detected symptomatically at a given age. (3) shorter intervals between attended screening appointments leads to less advanced cancer when detected by screening at any given age.

Following detection of a cancer, a woman is assigned a specific type of breast cancer, either DCIS or invasive and, for invasive cancers, a TNM stage (1 to 4) is assigned. This is done by sampling from the distribution of cancer types by age and mode of detection, found in the 2019 National Audit of Breast Cancer in Older Patients^24^ and summarised in Appendix Table 11. This show that older age and symptomatic detection are associated with a lower probability of having DCIS at diagnosis and, for invasive cancers, a higher probability of advanced cancer stages.

Appendix Table 11: Stage distribution of cancers by age and mode of detection under the current screening programme

| **Method of diagnosis and age** | **DCIS** | **Stage 1** | **Stage 2** | **Stage 3** | **Stage 4** |
| --- | --- | --- | --- | --- | --- |
| **Symptomatically detected** |  |  |  |  |  |
| age 20-54 | 6.1% | 29.2% | 46.5% | 13.0% | 5.2% |
| age 55-59 | 5.2% | 28.9% | 46.0% | 13.2% | 6.6% |
| age 60-64 | 5.1% | 28.4% | 46.3% | 12.8% | 7.4% |
| age 65-69 | 4.9% | 30.3% | 44.5% | 11.8% | 8.4% |
| age 70-74 | 4.7% | 30.3% | 44.6% | 11.7% | 8.8% |
| age 75-79 | 4.0% | 28.2% | 46.9% | 12.1% | 8.7% |
| age 80-84 | 3.6% | 27.0% | 47.7% | 12.3% | 9.4% |
| age 85 and above | 3.5% | 24.6% | 48.9% | 13.7% | 9.5% |
| **Screen detected** |  |  |  |  |  |
| age 20-54 | 24.6% | 47.3% | 23.8% | 3.7% | 0.6% |
| age 55-59 | 21.8% | 51.6% | 22.6% | 3.3% | 0.7% |
| age 60-64 | 18.9% | 56.1% | 21.4% | 3.0% | 0.7% |
| age 65-69 | 17.7% | 57.9% | 21.1% | 2.6% | 0.6% |
| age 70-74  (no screening programmes continue after 74 years of age) | 17.2% | 58.4% | 20.9% | 2.8% | 0.7% |

Appendix Table 11 shows the cancer type distribution for the current English national breast cancer screening programme, which offers triennial screening. The chance of detecting DCIS cancers and lower stage invasive cancer will be greater than in current national screening programme when screening intervals are shorter than three years. To account for this, when assigning cancer type at cancer detection the values in Appendix Table 11 are adjusted by duration of time between screen detection of cancer and her previous attended screening appointment. Evidence from the Breast Cancer Surveillance Consortium^38^ suggests that biennial and annual screening are associated with 19.6% and 32.5% relative increases in the proportion of DCIS cancers compared to triennial screening, respectively. As the BRAIDS screening regimen has an 18-month screening interval, the mean average increase between biennial and annual (25.5%) is applied.

To allow non-attendance to a scheduled screening appointment to be represented as a reduction in screening frequency the following adjustments were made to the distribution of screen detected cancer types: relative increases in DCIS for annual, 18-month screening and biennial screening were applied in the model if the duration of time since last attended screening was of 1.39 years or less, between 1.4 and 1.6 years and between 1.7 and 2.5 years, respectively. For durations larger than 2.5 years since last attended screening appointment the chance of the tumour being assigned to be DCIS was that observed under the current screening programme. For invasive cancers the shift in the assigned cancer to less advanced cancers are taken from the same source and presented in Appendix Table 12. As shown in the table, these adjustments are also applied based on duration since last attended screening. This is to account for the effect that repeated non-attendance increases the length of time a tumour can grow undetected, which will increase the probability the cancer is at an advanced stage at screen detection. We assume that a duration of time since last attended screening which is longer than under current national screening programme (three years) has the same distribution profile of screen detected cancer types as the current national screening programme (shown in Appendix Table 11).

Appendix Table 12: Summary of the assumed change in the distribution of cancers from the current UK screening programme with different screening intervals

| **Time since last screen in the model** | **Stage shift modifiers*** |
| --- | --- |
| Less than 1.39 years (represents annual screening) | 32.17% increase of DCIS |
| 1.4 to 1.6 years (represents 18-month screening) | 25.89% increase of DCIS |
| 1.6 to 2.5 years (represents biennial screening) | 19.6% increase of DCIS |
| 2.5 years to 9.9 years (represents 3-yearly screening) | None |
| Less than 1.5 years | 11.18% increase chance of the invasive tumour being stage 1  13.47% decrease chance of the invasive tumour being stage 2  6.07% decrease chance of the invasive tumour being stage 3 or 4 |
| Between 1.6 years and 2.5 years | 6.32% increase chance of the invasive tumour being stage 1  5.45% decrease chance of the invasive tumour being stage 2  2.17% decrease chance of the invasive tumour being stage 3 or 4 |
| Between 2.5 and 10 years | The current screening programme distribution of cancer types (see Appendix Table 8) |
| 10 years or greater (represents no screening) | Use stage distribution of symptomatic detection |

* Percentage change in the probability of cancer type for a screen detected cancer compared to that in the current screening programme (which is found in Appendix Table 11).

**Section 6 – cancer treatment costs**

All the costs reported in section 5 and section 6 are in 2019/2020 UK costs. Where published costs are for earlier years, they were inflated using the Hospital and Community Health Services Pay and Price Inflation Index (NHSCII prices) available in Unit Costs of Health and Social Care 2020^54^. Cancer treatment costs are based on total NHS costs incurred in each year since cancer detection (over a nine-year period) which vary by cancer type (DCIS or invasive cancer stages), age and mode of cancer detection (screen detected or symptomatically detected). The first-year total costs of stage 1 and stage 2 invasive breast cancer care were obtained from Sun and colleagues^44^ who use patient-level data to provide up-to-date estimates of early invasive breast cancer care costs by stage in England and to explore to what extent these costs varied based on patients’ ages and mode of detected (screen detected or symptomatically detected). The costs in the study were in 2016/2017 prices and uplifted to 2019/2020 prices based on hospital cost inflation over the three-year period of 6.24%. The results of a Log-Gamma multivariable analysis in 2019/2920 prices are presented in Appendix Table 13.

Appendix Table 13: Costs of stage 1 and stage 2 invasive cancer in the first year.

| Age | Stage 1 | Stage 2 | Stage 1 | Stage 2 | Mean of stage 1 and stage 2 | Mean of stage 1 and stage 2 |
| --- | --- | --- | --- | --- | --- | --- |
|  | Symptomatically detected | Symptomatically detected | Screen detected | Screen detected | Symptomatically detected | Screen detected |
| <60 | £10,074 | £12,106 | £9,679 | £11,710 | £11,090 | £10,695 |
| 60-69 | £8,937 | £10,968 | £8,541 | £10,573 | £9,952 | £9,557 |
| 70-79 | £7,591 | £9,622 | £7,195 | £9,227 | £8,606 | £8,211 |
| 80-89 | £4,984 | £7,015 | £4,588 | £6,620 | £5,999 | £5,604 |
| 90+ | £2,653 | £4,684 | £2,258 | £4,289 | £3,668 | £3,273 |

Treatment costs are stage 3 and stage 4 and in years after detection were established by combining the information in Appendix Table 13 with cost estimates from Laudicella *et al* (2016)^45^. This is a UK retrospective cohort study that observed total treatment costs for over 350,000 patients in England diagnosed with breast cancer.

The first-year cost of cancer care in stage 3 and stage 4 when screen or symptomatically detected were estimated by calculating the proportionate increase in total treatment costs for stage 3 and stage 4 cancers from Laudicella^45^ to the mean first year total costs for stage 1 and stage 2 breast cancer (as shown in Appendix Table 13) by mode of detection established from Sun^44^. Cancer follow up treatment costs vary over nine years post cancer detection and these costs were established by applying the proportionate decrease in total cancer care costs in each subsequent year from cancer detection (compared to first year costs) taken from Laudicella^45^. The calculations applied to establish the proportionate changes in costs and their values are presented in Appendix Table 14.  In addition to establishing follow up costs after the first-year cancer detection, the proportionate changes in Appendix Table 14 are also applied to the Sun^44^ first-year costs to calculate stage 3 and 4 total costs. The resulting total invasive cancer care costs over nine years by age, stage and mode of detection are presented in Appendix Table 15.

Appendix Table 14: Proportionate changes in total costs (costs in 2010 prices) in comparison to stage 1/stage 2 invasive cancer in the first year for more advanced stages and subsequent years of follow up.

| Years after diagnosis | Stages 1–2 | Stages 1–2 | Stages 3–4 | Stages 3–4 | Proportionate change in subsequent years from 1st year | Proportionate change in subsequent years from 1st year | Proportionate change in stage 3-4 from stage 1- 2 | Proportionate change in stage 3-4 from stage 1-2 |
| --- | --- | --- | --- | --- | --- | --- | --- | --- |
|  | Ages 18–64 | Ages 65 or more | Ages 18–64 | Ages 65 or over | Ages 18–64 | Ages 65 or over | Age 18–64 | Age 65 or over |
| 1 | £10,746 | £7,597 | £13,315 | £8,804 | - | - | 1.2391 | 1.1589 |
| 2 | £3,357 | £2,529 | £5,785 | £3,650 | 0.3124 | 0.3329 | 1.7233 | 1.4433 |
| 3 | £1,953 | £2,156 | £3,782 | £3,170 | 0.1817 | 0.2838 | 1.9365 | 1.4703 |
| 4 | £1,627 | £2,230 | £2,932 | £2,924 | 0.1514 | 0.2935 | 1.8021 | 1.3112 |
| 5 | £1,617 | £2,077 | £2,841 | £2,957 | 0.1505 | 0.2734 | 1.7570 | 1.4237 |
| 6 | £1,547 | £2,174 | £2,645 | £2,783 | 0.1440 | 0.2862 | 1.7098 | 1.2801 |
| 7 | £1,394 | £2,063 | £2,618 | £2,903 | 0.1297 | 0.2716 | 1.8780 | 1.4072 |
| 8 | £1,376 | £2,134 | £2,559 | £2,454 | 0.1280 | 0.2809 | 1.8597 | 1.1500 |
| 9 | £1,279 | £2,204 | £1,848 | £2,932 | 0.1190 | 0.2901 | 1.4449 | 1.3303 |

Appendix Table 15: Costs of risk assessment and invasive cancer care

| **Year after cancer detection** | **TNM Stage 1–2** | | | | | **TNM Stage 3–4** | | | | |
| --- | --- | --- | --- | --- | --- | --- | --- | --- | --- | --- |
| Screen detected cancer: | Ages 18–60 | Ages 60-69 | Ages 70-79 | Ages 80-89 | Ages 90+ | Ages 18–60 | Ages 60-69 | Ages 70-79 | Ages 80-89 | Ages 90+ |
| Year 1 | £10,695 | £9,557 | £8,211 | £5,604 | £3,273 | £13,346 | £11,936 | £9,578 | £6,557 | £3,856 |
| Year 2 | £3,341 | £2,986 | £2,733 | £1,866 | £1,090 | £7,185 | £6,426 | £4,602 | £3,150 | £1,853 |
| Year 3 | £1,944 | £1,737 | £2,330 | £1,590 | £929 | £4,697 | £4,201 | £3,997 | £2,736 | £1,609 |
| Year 4 | £1,619 | £1,447 | £2,410 | £1,645 | £961 | £3,641 | £3,257 | £3,687 | £2,524 | £1,484 |
| Year 5 | £1,609 | £1,438 | £2,245 | £1,532 | £895 | £3,528 | £3,156 | £3,728 | £2,552 | £1,501 |
| Year 6 | £1,540 | £1,376 | £2,350 | £1,604 | £937 | £3,285 | £2,938 | £3,509 | £2,402 | £1,413 |
| Year 7 | £1,387 | £1,240 | £2,230 | £1,522 | £889 | £3,251 | £2,908 | £3,660 | £2,506 | £1,473 |
| Year 8 | £1,369 | £1,224 | £2,306 | £1,574 | £919 | £3,178 | £2,842 | £3,094 | £2,118 | £1,246 |
| Year 9 | £1,273 | £1,137 | £2,382 | £1,626 | £950 | £2,295 | £2,053 | £3,697 | £2,531 | £1,488 |
| Symptom detected cancer: |  |  |  |  |  |  |  |  |  |  |
| Year 1 | £11,090 | £9,952 | £8,606 | £5,999 | £3,668 | £13,741 | £12,331 | £9,973 | £6,952 | £4,251 |
| Year 2 | £3,464 | £3,109 | £2,865 | £1,997 | £1,221 | £7,397 | £6,638 | £4,792 | £3,340 | £2,042 |
| Year 3 | £2,016 | £1,809 | £2,442 | £1,703 | £1,041 | £4,836 | £4,340 | £4,162 | £2,901 | £1,774 |
| Year 4 | £1,679 | £1,507 | £2,526 | £1,761 | £1,077 | £3,749 | £3,365 | £3,839 | £2,676 | £1,636 |
| Year 5 | £1,669 | £1,498 | £2,353 | £1,640 | £1,003 | £3,633 | £3,260 | £3,882 | £2,706 | £1,655 |
| Year 6 | £1,597 | £1,433 | £2,463 | £1,717 | £1,050 | £3,382 | £3,035 | £3,654 | £2,547 | £1,557 |
| Year 7 | £1,439 | £1,291 | £2,337 | £1,629 | £996 | £3,348 | £3,004 | £3,811 | £2,657 | £1,624 |
| Year 8 | £1,420 | £1,274 | £2,417 | £1,685 | £1,030 | £3,272 | £2,937 | £3,222 | £2,246 | £1,373 |
| Year 9 | £1,320 | £1,185 | £2,497 | £1,740 | £1,064 | £2,363 | £2,121 | £3,849 | £2,683 | £1,641 |

The costs for DCIS treatment were derived from the costs of treating invasive cancer based on the ratio of cancer stage 0 to stage 1-2 costs reported in Blumen, Fitch and Polkus^55^. This publication provided separate ratios for the first and second year of treatment; the latter ratio was assumed to hold for all subsequent years. The cost multiplier (the ratio of DCIS treatment costs to stage 1/stage 2 invasive cancer costs) was applied to the costs of care for stage 1/stage 2 invasive cancer by age, duration since cancer detection and mode of detection (Table 5 in the main text of the paper). This publication provided separate ratios for the first and second year of treatment; the latter ratio was assumed to hold for all subsequent years. The multiplier applied in the first year was 0.738 and in subsequent years it is 0.730. The resulting DCIS treatment costs are presented in Appendix Table 16.

Appendix Table 16: Costs of DCIS cancer care by age, mode of detection and duration since detection.

| Year after cancer detection | Screen detected | | | | | Symptomatically detected | | | | |
| --- | --- | --- | --- | --- | --- | --- | --- | --- | --- | --- |
|  | Ages 18–60 | Ages 60-69 | Ages 70-79 | Ages 80-89 | Ages 90 | Ages 18–60 | Ages 60-69 | Ages 70-79 | Ages 80-89 | Ages 90 or over |
| 1 | £7,897 | £7,057 | £6,063 | £4,138 | £2,417 | £8,189 | £7,349 | £6,355 | £4,430 | £2,709 |
| 2 | £2,440 | £2,181 | £1,997 | £1,363 | £796 | £2,531 | £2,271 | £2,093 | £1,459 | £892 |
| 3 | £1,420 | £1,269 | £1,702 | £1,162 | £678 | £1,472 | £1,321 | £1,784 | £1,244 | £760 |
| 4 | £1,183 | £1,057 | £1,760 | £1,202 | £702 | £1,226 | £1,101 | £1,845 | £1,286 | £787 |
| 5 | £1,175 | £1,050 | £1,640 | £1,119 | £654 | £1,219 | £1,094 | £1,719 | £1,198 | £733 |
| 6 | £1,125 | £1,005 | £1,716 | £1,171 | £684 | £1,166 | £1,046 | £1,799 | £1,254 | £767 |
| 7 | £1,013 | £906 | £1,629 | £1,112 | £649 | £1,051 | £943 | £1,707 | £1,190 | £728 |
| 8 | £1,000 | £894 | £1,685 | £1,150 | £672 | £1,037 | £931 | £1,766 | £1,231 | £753 |
| 9 | £930 | £831 | £1,740 | £1,188 | £694 | £964 | £865 | £1,824 | £1,271 | £777 |

**Section 7 – cancer screening costs**

Where available, the costs of screening instruments were obtained from 2019/2020 NHS reference costs^57^. A summary of the unit costs, sources and costing assumptions are provided in Appendix Table 17.

The most recently available mammography costs were from NHS reference costs 2005/06 (£40, Band B1 – Mammography) and after accounting for inflation (using Hospital and Community Health Services Pay and NHSCII price indices^54^) this amounted to a cost of £57.69. As no reference costs were available for contrast-enhanced spectral mammography, this imaging technique was assumed to be 9.44% more expensive than standard mammography^58^ giving a cost of £63.14. The cost for MRI (£148.24) was based on the code of Magnetic Resonance Imaging Scan of One Area, without Contrast, 19 years and over (RD01A). Abbreviated MRI cost (118.68) was assumed to be the lowest of the available MRI cost tariffs: Magnetic Resonance Imaging Scan of One Area, with Post-Contrast Only, 19 years and over (HRG code: RD02A). The Post-contrast tariff was applied because breast cancer lesion detection with MRI is primarily performed by using postcontrast images (Mann, Cho and Moy, 2019). The cost of 3D ultrasound was (£88.31) taken from computerised Tomography Scan of One Area, without Contrast, 19 years and over (HRG code: RD20A). The cost of mobile ultrasound (£61.18) was tariff: Ultrasound Scan Mobile or Intraoperative Procedures, with duration of less than 20 minutes (HRG code: RD44Z).

All women with positive diagnosis of cancer, whether detected symptomatically and at a screening appointment, incur a cost due to recall for further assessment of £290.87. The cost is based on the use of mammography and mobile ultrasound (less than 20 minutes), as stated in NHS guidance on the instruments used in further assessment (PHE, 2016). Additional costs at further assessment include core biopsy and open biopsy which are incurred by 47.5% and 1.1% of women respectively, based on the latest national screening programme activity data^1^. Cost of core biopsy (£353.50) is from breast surgery outpatient procedures NHS reference cost YJ03Z: Biopsy of Lesion of Breast and Associated Lymph Nodes. The cost of an open biopsy (£294.00) is assumed to be the mean of two tariff costs for breast surgery outpatient procedures: YJ09Z (Vacuum Assisted Biopsy of Lesion of Breast: £410.40) and YJ10Z (Insertion of, Wire or Marker, for Localisation of Breast Lesion: £177.60). 0.8% of women have a further short-term recall in the NBSCP^25^ when definitive diagnosis could not be made after further assessment procedures. The cost of this was £104.81 based on the 2019/2020 National Tariff Payment System currency code (CMDT-B) for a breast cancer multidisciplinary team meeting.

The implementation of personalised screening strategies incurred a cost for risk assessment. The cost of a standard risk assessment (without polygenetic risk) was £11.54 based on the cost reported in previously published UK cost-effectiveness analysis^43^ when inflated to 2019/2020 prices. It was assumed that augmenting this cost with polygenetic risk would incur a saliva test which has been reported to be £53.23 in 2019/2020 prices^57^. In addition to this cost, all risk assessments include a mammography screen at a cost of £57.69. Women who did not attend a scheduled risk assessment screening appointment were assumed to incur 10% of the cost of a routine (without polygenetic risk) risk assessment (£1.54). Women with cancers detected symptomatically incur a cost of £39.00 which is the cost of surgery consultation lasting 9.22 minutes with a General practitioner in the Unit Costs of Health & Social Care 2020^54^. The cancer costs for treatment by stage and time since cancer detection are based on an observational studies of total NHS costs observed in hospitals post treatment, and therefore include monitoring and screening costs for recurrence after a cancer has been detected.

Appendix Table 17: Screening and risk assessment costs (2019/29).

| **Instrument** | **Cost (£)** | **Source and costing assumptions** |
| --- | --- | --- |
| Mammography | £57.69 | The £40 mammography cost in 2005/06 reference cost (Band B1) was adjusted using the RPI from Jan 2006 to Jan 2009. The cost was further adjusted to represent inflated from 2009/10 to 2015/2016 using the hospital and community health services (HCHS) pay and price inflation index, and then adjusted using NHSCII prices for inflation in the period 2015/2016 to 2019/20. |
| Contrast-enhanced spectral mammography | £63.14 | There has not been a published NHS reference cost for CEM. The relative increase in cost of delivering a contrast-enhanced spectral mammography compared to digital mammography is an increase of 9.44% in costs, taken from a US source^58^ |
| Magnetic resonance imaging (full) | £148.24 | Magnetic Resonance Imaging Scan of One Area, without Contrast, 19 years and over (RD01A) |
| Magnetic resonance imaging (abbreviated) | £118.68 | Assumed to be the lowest MRI cost tariff: Magnetic Resonance Imaging Scan of One Area, with Post-Contrast Only, 19 years and over (HRG code: RD02A): £118.68 |
| Ultrasound scan: 3D (automated) | £88.31 | Computerised Tomography Scan of One Area, without Contrast, 19 years and over (HRG code: RD20A): £88.31 |
| Ultrasound scan: mobile (supplemental) | £61.18 | Ultrasound Scan, Mobile or Intraoperative Procedures, with duration of less than 20 minutes (HRG code: RD44Z): £61.18 |
| Further assessment (after a positive diagnosis) | £290.87 | 47.5% of women who are referred undergo core biopsy (NHS BSP) and 1.1% of woman have an open biopsy as reported in the Breast Screening Programme England, 2019-20 report^25^ (Page 24).  Cost of core biopsy is from breast surgery outpatient procedures NHS reference cost YJ03Z: Biopsy of Lesion of Breast and Associated Lymph Nodes. £353.50  The cost of an open biopsy is assumed to be the mean of two tariff costs for breast surgery outpatient procedures: YJ09Z (Vacuum Assisted Biopsy of Lesion of Breast: £410.40) and YJ10Z (Insertion of, Wire or Marker, for Localisation of Breast Lesion: £177.60)  Therefore, the biopsy cost is: (0.475 x 353.50) + (0.011 x ((410.40 + 177.60) % 2) = £171.15  Among women recalled for assessment, the model assumes that all referrals will undergo a further mammography (£57.69) and ultrasound scan (£61.18); and additional cost of £118.88. These assumptions are supported by NHS best practice guidance (2016) for imagining (recall) procedures that are a result of a mammographic abnormality.  0.8% of women referred for assessment were recommended for short term recall (page 25 of the 2019/2020 report). This represents as small minority of cases, referred for assessment, where a definitive diagnosis cannot be made in recall assessment. Short term recall is given a unit cost of £104.81 based on reference cost currency code CMDT-B (Breast Cancer MDT Meetings)  Therefore, the management of women recalled for assessment is: £171.15(biopsy cost) + 118.88 (additional screening instrument cost) + (£104.81 x 0.008: short term recall cost) = 290.87 |
| Risk assessment: with / without saliva test | £122.46/  £69.23 | The cost of assigning TC without genetic test is based on expert opinion in Table 3 from a UK source^43^. This is an estimate of the cost of administering risk and breast density–based stratification was made on the basis of a nurse visit, Data entry (grade 4 admin), Consultant led risk counselling, postage and stationery. It is £10.57 in 2014/2015 prices, and 2019/2020 NHSCII prices (taken from PSSRU 2019/2020), the cost is £11.54  I assume a salvia test costs £53.23, which is the £50 cost estimate for genotyping in a UK source^57^ uplifted from 2016/2017 prices (when the analysis in that paper took place) to 2019/20 prices. All risk assessments also include a mammography screen (£57.69). |
| Costs for activities involved in screening: risk assessment invitation without attendance (i.e. non-response to an invitation) | £1.54 | Assumed to be 10% of the cost of a routine risk assessment. Thus, £11.54 x 0.1 = £1.54 |
| Cost of attending a GP session for woman who had a cancer detected symptomatically (rather than at a screening appointment) | £39.00 | Per surgery consultation lasting 9.22 minutes with a general practitioner (PSSRU, 2019/2020)^54^ |

**Section 8 - Health losses**

Mammography and contrast-enhanced spectral mammogram incur pain represented by a utility loss of 0.072, which is the mean disutility found in a systematic review^59^, and is assumed to last for one week, hence generating a QALY loss of 0.0014. We assume this utility loss also occurs after a true or FP finding, due to the use of mammography in investigative work up. We assume any utility loss for potential complications or problems with other forms of investigative work up (such as biopsies) after a positive finding is accounted for by disutility with treatment. FPs have a QALY loss of 0.077, based on a utility loss of 0.05 in the first month and 0.025 for the next 35 months^60^. Age-specific EQ5D-3L utility values for women without a diagnosis of breast cancer were taken from women in the general population in England^61^. Post-cancer treatment disutility values were applied to these values to estimate age-specific post-cancer health utility. The treatment disutility values were taken from a recent study that found decrements of 0.098 (stage 1), 0.099 (stage 2), 0.118 (stage 3), 0.186 (stage 4)^62^. Post-treatment health utility was found to be higher by 0.038, which is introduced into the model by assuming post-treatment begins after 3 months (stage 1), 4.5 months (stage 2), 9 months (stage 3) or never occurs (stage 4)^63^. Based on evidence from UK women with breast cancer there is no disutility from non-metastatic cancer 60 months after its detection^64^. There is an absence of published health utility values for DCIS breast cancer, and therefore Stage 1 invasive cancer utility values are applied as the cancer treatments are similar^24^. A summary of utility values is provided in the below Appendix Table 18.

Appendix Table 18: Health utility and QALY decrements

| **Health utility and QALY decrements** | **DCIS and Stage 1** | **Stage 2** | **Stage 3** | **Stage 4** |
| --- | --- | --- | --- | --- |
| Months after cancer detection: |  |  |  |  |
| Up to 3 months | 0.098 | 0.099 | 0.118 | 0.186 |
| Between 3 and 4.5 months | 0.060 | 0.099 | 0.118 | 0.186 |
| Between 4.5 and 9 months | 0.060 | 0.061 | 0.118 | 0.186 |
| Between 9 and 59 months | 0.060 | 0.061 | 0.080 | 0.186 |
| 60 months and after | No decrement | No decrement | No decrement | 0.186 |
| QALY loss from a mammogram screen | 0.0014 | | | |
| QALY loss from FP | 0.0771 | | | |

FP: False positive. QALY: Quality adjusted life-year

**Section 9 – cancer survival parameters**

Appendix Table 19: Hazard ratios for invasive cancer survival

| **Cancer survival risks** | **Hazard Ratio** |
| --- | --- |
| TNM stage 1 | 1 (general population survival) |
| TNM stage 2 | 3.39 |
| TNM stage 3 | 11.96 |
| TNM stage 4 | 30.05 |
| Age at cancer detection |  |
| 50-69 | 1 |
| 70-74 | 1.49 |
| 75-79 | 1.36 |
| 80+ | 1.23 |
| Screen detected (compared to symptomatically detected) | 0.66 |

We assume the survival disadvantage for Stage 1 cancer is the same as DCIS. This is based on an analysis of the NHSBSP data which found that the 15-year relative survival was not statistically significant from the general population^42^.

**Section 10 –sensitivity analysis**

Appendix Table 20: Scenario analyses part a: Net health benefit at a willingness to pay of £20,000

| Intervention | Starting age at age 35 | Disc. rate 1.5% | No screening disutilities | Screening disutility doubled | Screening sen. increased by 10% | Screening sen. decreased by 10% |
| --- | --- | --- | --- | --- | --- | --- |
| No screening | 18.8396 | 21.8699 | 16.5538 | 16.5534 | 16.5466 | 16.5466 |
| PROCAS | 18.8721 | 21.9893 | 16.6286 | 16.5937 | 16.8293 | 16.6357 |
| ASSURE 1 | 18.8480 | 21.9512 | 16.6078 | 16.5752 | 16.5966 | 16.5938 |
| ASSURE 2 | 18.8448 | 21.9415 | 16.6019 | 16.5676 | 16.5902 | 16.5871 |
| ASSURE 3 | 18.8256 | 21.9084 | 16.5792 | 16.5417 | 16.5646 | 16.5601 |
| ASSURE 4 | 18.8382 | 21.9272 | 16.5929 | 16.5507 | 16.5768 | 16.5725 |
| BRAIDS 2 | 18.8445 | 21.9435 | 16.6014 | 16.5629 | 16.5921 | 16.5853 |
| BRAIDS 3 | 18.8426 | 21.9419 | 16.6048 | 16.5564 | 16.5888 | 16.5858 |
| BRAIDS 4 | 18.8430 | 21.9383 | 16.5975 | 16.5569 | 16.5854 | 16.5807 |
| Current screening | 18.8219 | 21.9087 | 16.5784 | 16.5497 | 16.5676 | 16.5647 |

Breast Screening Risk Adaptive Imaging for Density (BRAID), Adapting Breast Cancer Screening Strategy Using Personalised Risk Estimation (ASSURE), Predicting Risk of Cancer at Screening (PROCAS).

Appendix Table 21: Scenario analyses part b: Net health benefit at a willingness to pay of £20,000

| Intervention | Same FP rate for all screening | Cancer treatment costs increased 10% | Cancer treatment costs decreased 10% | HRQoL loss from cancer increased by 10% | HRQoL loss from cancer decreased by 10% |
| --- | --- | --- | --- | --- | --- |
| No screening | 16.5536 | 16.5476 | 16.5596 | 16.5496 | 16.5576 |
| PROCAS | 16.6112 | 16.6037 | 16.6187 | 16.6071 | 16.6152 |
| ASSURE 1 | 16.5915 | 16.5850 | 16.5979 | 16.5880 | 16.5949 |
| ASSURE 2 | 16.5848 | 16.5784 | 16.5911 | 16.5813 | 16.5882 |
| ASSURE 3 | 16.5656 | 16.5541 | 16.5668 | 16.5569 | 16.5639 |
| ASSURE 4 | 16.5781 | 16.5654 | 16.5782 | 16.5684 | 16.5752 |
| BRAIDS 2 | 16.5822 | 16.5758 | 16.5886 | 16.5787 | 16.5856 |
| BRAIDS 3 | 16.5815 | 16.5742 | 16.5870 | 16.5773 | 16.5839 |
| BRAIDS 4 | 16.5783 | 16.5708 | 16.5836 | 16.5738 | 16.5805 |
| Current screening | 16.5641 | 16.5577 | 16.5704 | 16.5605 | 16.5677 |

Breast Screening Risk Adaptive Imaging for Density (BRAID), Adapting Breast Cancer Screening Strategy Using Personalised Risk Estimation (ASSURE), Predicting Risk of Cancer at Screening (PROCAS).

Appendix Table 22: Scenario analyses part c: No within stage survival benefit from screening

| **Regimen** | **Undiscounted values** | | | **Disc. Costs** | **Disc. QALYs** | **NHB** |
| --- | --- | --- | --- | --- | --- | --- |
|  | **Total cost** | **Total QALYs** | **Life years** |  |  |  |
| No screening | £2,411 | 27.931 | 35.809 | £1,188 | 16.613 | 16.554 |
| PROCAS | £3,298 | 28.156 | 36.035 | £1,948 | 16.698 | 16.601 |
| ASSURE 1 | £3,179 | 28.130 | 36.035 | £1,839 | 16.684 | 16.592 |
| ASSURE 2 | £3,192 | 28.097 | 35.999 | £1,851 | 16.669 | 16.576 |
| ASSURE 3 | £3,328 | 28.076 | 36.016 | £1,940 | 16.654 | 16.557 |
| ASSURE 4 | £3,444 | 28.102 | 36.024 | £2,032 | 16.668 | 16.567 |
| BRAID 2 | £3,464 | 28.135 | 36.044 | £2,076 | 16.684 | 16.580 |
| BRAID 3 | £3,359 | 28.121 | 36.036 | £2,002 | 16.676 | 16.576 |
| BRAID 4 | £3,589 | 28.126 | 36.035 | £2,170 | 16.681 | 16.572 |
| Current screening | £3,076 | 28.066 | 36.008 | £1,736 | 16.650 | 16.564 |

Appendix Table 23: Probabilistic sensitivity analysis results of RSBCS programmes

| **Regimen** | **Undiscounted values** | | | **Disc. costs** | **Disc. QALYs** | **NHB** |
| --- | --- | --- | --- | --- | --- | --- |
|  | **Total cost** | **Total QALYs** | **Life years** |  |  |  |
| No screening | £2,405 | 27.971 | 35.860 | £1,183 | 16.624 | 16.565 |
| PROCAS | £3,322 | 28.220 | 36.126 | £1,971 | 16.718 | 16.619 |
| ASSURE 1 | £2,791 | 28.125 | 36.058 | £1,632 | 16.680 | 16.599 |
| ASSURE 2 | £2,818 | 28.114 | 36.056 | £1,646 | 16.674 | 16.592 |
| ASSURE 3 | £2,938 | 28.074 | 36.038 | £1,733 | 16.653 | 16.566 |
| ASSURE 4 | £3,046 | 28.107 | 36.059 | £1,816 | 16.669 | 16.578 |
| BRAID 2 | £3,087 | 28.138 | 36.079 | £1,869 | 16.685 | 16.591 |
| BRAID 3 | £2,986 | 28.128 | 36.079 | £1,796 | 16.677 | 16.588 |
| BRAID 4 | £3,216 | 28.136 | 36.080 | £1,964 | 16.683 | 16.585 |
| Current screening | £2,683 | 28.061 | 36.026 | £1,527 | 16.648 | 16.572 |

Appendix Table 24: Comparison of base case and probabilistic sensitivity analysis ICERs

| **Regimen** | **Deterministic results** | | | **Probabilistic results** | | |
| --- | --- | --- | --- | --- | --- | --- |
|  | **Disc. costs** | **Disc. QALYs** | **ICER compared to the UK NHSBSP** | **Disc. costs** | **Disc. QALYs** | **ICER compared to UK NHSBSP** |
| No screening | £1,188 | 16.613 | Current screening dominates | £1,183 | 16.624 | Current screening dominates |
| PROCAS | £1,971 | 16.710 | £6,301 | £1,971 | 16.718 | £5,640 |
| ASSURE 1 | £1,641 | 16.673 | £3,184 | £1,632 | 16.680 | £2,405 |
| ASSURE 2 | £1,649 | 16.667 | £4,244 | £1,646 | 16.674 | £3,280 |
| ASSURE 3 | £1,743 | 16.648 | £30,977 | £1,733 | 16.653 | £16,406 |
| ASSURE 4 | £1,825 | 16.663 | £12,984 | £1,816 | 16.669 | £9,887 |
| BRAID 2 | £1,876 | 16.676 | £9,670 | £1,869 | 16.685 | £7,570 |
| BRAID 3 | £1,804 | 16.671 | £8,930 | £1,796 | 16.677 | £7,096 |
| BRAID 4 | £1,972 | 16.676 | £12,476 | £1,964 | 16.683 | £10,164 |
| Current screening | £1,537 | 16.641 |  | £1,527 | 16.648 |  |

Appendix Table 25: Table of PSA parameter values

Unless otherwise stated, all distributions assumed that the standard error was 10% of the mean. Truncated Normal distributions were truncated to be positive (>0) unless otherwise stated.

| **Variable** | **Distribution** |
| --- | --- |
| Breast density by age | Dirichlet by age |
| Breast density change as women age | Normal |
| Overdiagnosis rate | Truncated Normal |
| Age of symptomatic detection under no screening | Log-normal |
| Relative increase in the incidence of cancer moving from screening every three years to two years | Normal (standard error 4.235 derived from (95% CI 0.8–17.4%))^14^ |
| QALY loss for experiencing mammography screening | Truncated Normal (standard error 0.0144)^59^ |
| QALY loss for experiencing FP | Truncated Normal |
| Excess risk of mortality 10 years post DCIS diagnosis | Log-normal |
| relative mortality rates for invasive cancer | Normal, truncated to be >1 with uncertainty based on confidence intervals in reference^39^ |
| Tumour presence time | Sampled from linear model |
| Average change in the percentage of DCIS detected by 2-yearly and 1-yearly compared to 3-yearly program | Normal |
| Invasive cancer utility decrements by stage and time since diagnosis | Truncated Normal |
| Screening costs | Truncated Normal |
| Cost of treating cancer | Log-normal |
| Invasive Tumour sizes | Log-normal |
| DCIS Tumour sizes | Log-normal |
| Digital mammography specificity | Coefficients varied using Normal distributions with standard error derived from 95% CI obtained from Table 2 of reference.^22^ |
| Relative improvement in sensitivity of non-mammography instruments | Normal |
| Handheld ultrasound sensitivity (reduction in comparative sensitivity relative to automated ultrasound) | Truncated Normal (standard error 4.235 derived from (95% CI 8.4–14.7%))^29^ |
| Sensitivity of abbreviated and full MRI | Two-step approach: beta(54, 60) for sensitivity under abbreviated MRI, to which a beta(1, 6) was added to reflect the additional sensitivity with full MRI^30^ |
| False-positive rates for instruments | Truncated Normal |

Appendix Figure 1. Cost effectiveness acceptability curve of RSBCS programmes


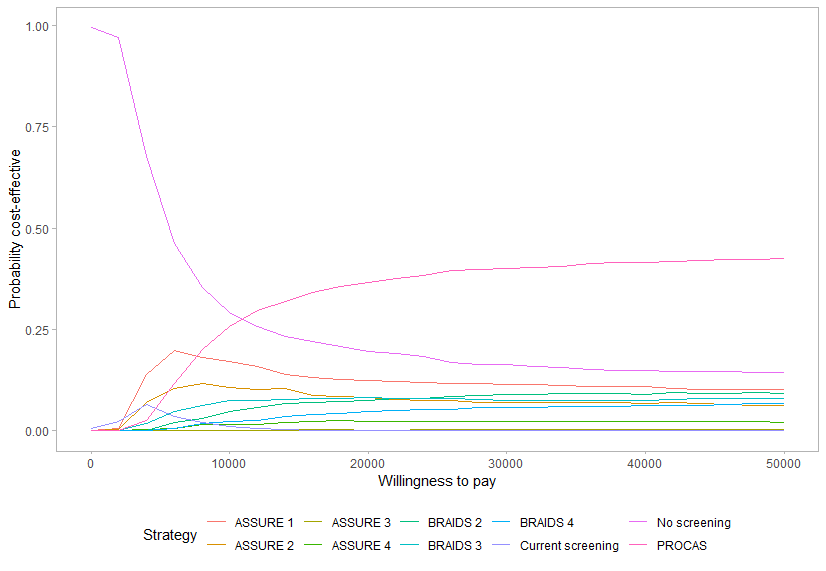


**References present in this online appendix but not the main manuscript**

50. Ciatto, S., Visioli, C., Paci, E. and Zappa, M.. Breast density as a determinant of interval cancer at mammographic screening. British journal of cancer, 90(2), 393-396 (2004).

51. Porter, P.L., El-Bastawissi, A.Y., Mandelson, M.T., Lin, M.G., Khalid, N., Watney, E.A., Cousens, L., White, D., Taplin, S. and White, E. Breast tumor characteristics as predictors of mammographic detection: comparison of interval-and screen-detected cancers. *Journal of the National Cancer Institute*, *91*(23), 2020-2028 (1999)

52. Perron, L., Chang, S.L., Daigle, J.M., Vandal, N., Theberge, I., Diorio, C., Lemieux, J., Pelletier, E. and Brisson, J.. Breast cancer subtype and screening sensitivity in the Quebec Mammography Screening Program. *Journal of medical screening*, *26*(3), 154-161 (2019)

53. Vourtsis, A. and Berg, W.A. Breast density implications and supplemental screening. *European radiology*, *29*(4).1762-1777 (2019)

54. Jones, K.C. and Burns, A., 2020. Unit costs of health and social care. (2020).

55. Blumen, H., Fitch, K., & Polkus, V. Comparison of treatment costs for breast cancer, by tumor stage and type of service. *American health & drug benefits*, **9**(1), 23 (2016).

56. National Schedule of NHS Costs 2019/2020. NHS England. Available at: <https://www.england.nhs.uk/publication/2019-20-national-cost-collection-data-publication/>

57. Pashayan, N., Morris, S., Gilbert, F. J., & Pharoah, P. D. Cost-effectiveness and benefit-to-harm ratio of risk-stratified screening for breast cancer: a life-table model. *JAMA oncology*, *4*(11), 1504-1510. (2018).

58. Patel, B. K., Gray, R. J., & Pockaj, B. A. Potential cost savings of contrast-enhanced digital mammography. *American Journal of Roentgenology*, *208*(6), 231-237 (2017)

59. Li, L., Severens, J. L., & Mandrik, O. Disutility associated with cancer screening programs: a systematic review. *PloS one* **14**(7), e0220148 (2019).

60. Brodersen, J., & Siersma, V. D. Long-term psychosocial consequences of false-positive screening mammography. *The Annals of Family Medicine* **11**(2), 106-115 (2013).

61. Ara, R., & Brazier, J. E. Populating an economic model with health state utility values: moving toward better practice. *Value in Health* **13**(5), 509-518 (2010)

62. Wang, L., Shi, J. F., Zhu, J., Huang, H. Y., Bai, Y. N., Liu, G. X. et al. Health-related quality of life and utility scores of patients with breast neoplasms in China: A multicenter cross-sectional survey. *The Breast* **39**, 53-62. (2018).

63. Health Partners. Breast cancer Treatment by Stage. <https://www.healthpartners.com/blog/breast-cancer-treatment-by-stage/>. Accessed 18^th^ March 2022

64. Schleinitz, M. D., DePalo, D., Blume, J., & Stein, M. Can differences in breast cancer utilities explain disparities in breast cancer care?. *Journal of general internal medicine* **21**(12), 1253-1260 (2006).
